# Supplementary material for: Learning about the Ellsberg Paradox reduces, but does not abolish, ambiguity aversion
Source: PLoS One. 2020 Mar 4;15(3):e0228782. doi: 10.1371/journal.pone.0228782 (PMC7055742; doi:10.1371/journal.pone.0228782)

S1 Text. Details of active calculation (AC) and non-active calculation (NC) intervention methods, and control (base rate) intervention method.

Active Calculation Condition

**Note: For this demonstration, text written in gray depends on responses and may be slightly different between participants. Also, the ambiguous lottery used for calculation is an example, and the actual one shown to the participant depends on her own choice in pre-intervention trials.*

Text written in yellow indicates direct input from participants, and the examples shown in this document indicate correct reponses.

Click on INCORRECT *buttons to see the feedback slide if the participant gives a wrong answer.*

Then click on RETURN *buttons to move on to the next slide if the participant eventually gives a correct answer.*

These two buttons are for demonstration only, and did not appear in the actual intervention.

In this experiment you have seen two types of lotteries. Those in which you know your exact chance of winning (like in the lottery on the left), and those in which you do not (like in the lottery on the right).

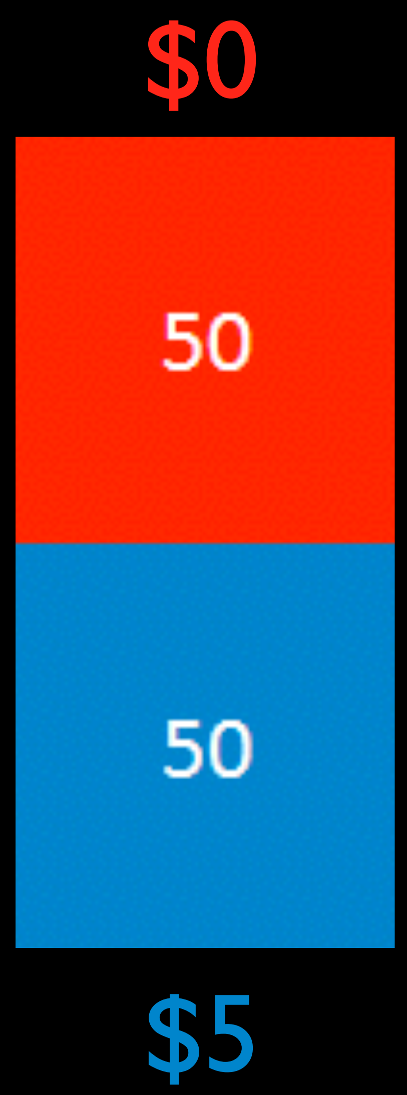

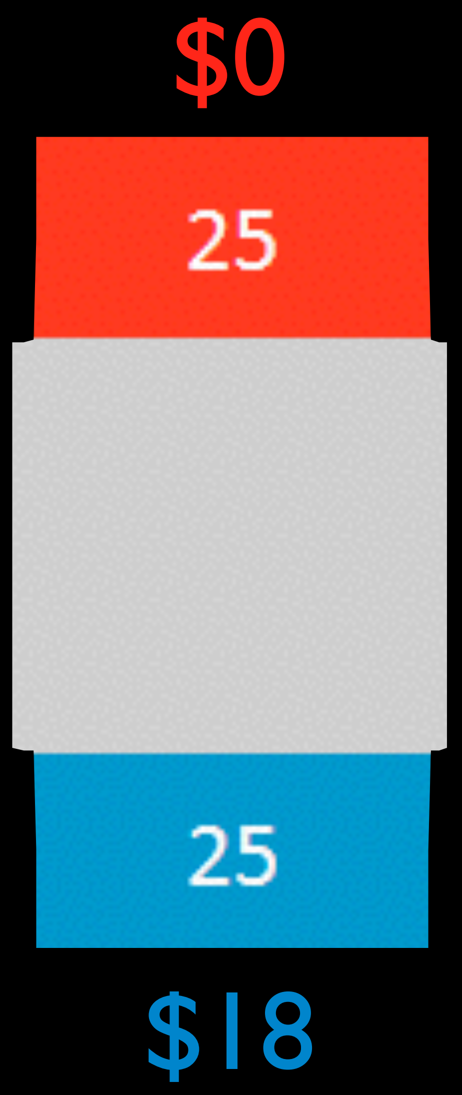

When people know their exact chance of winning different people make different choices. Some prefer taking low risks for small gains (like on the left) while others prefer taking high risks for large gains (like on the right). Choices on these kinds of lotteries are a matter of personal preference.

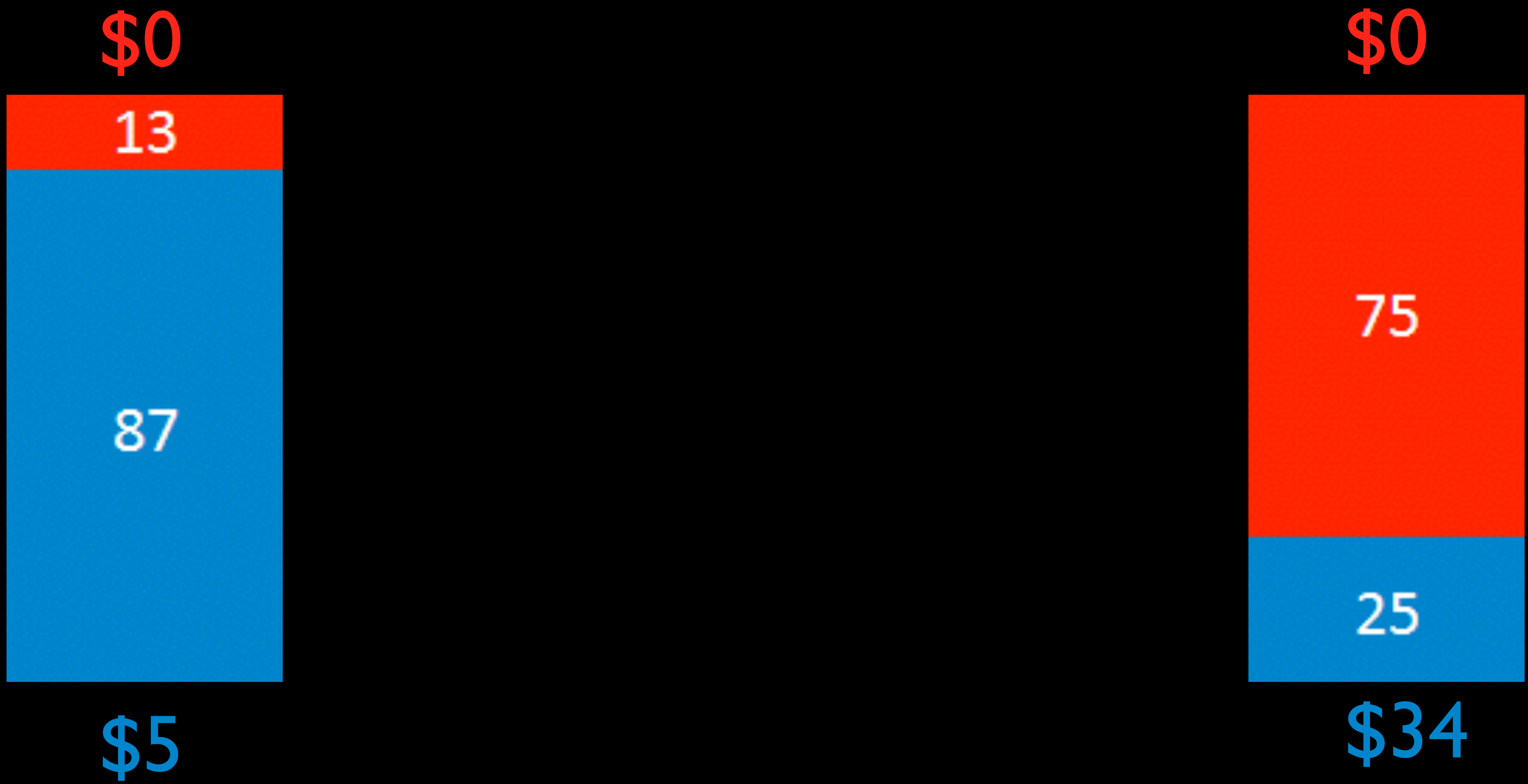

You were also presented with the same lottery, except the winning color was red instead of blue. Just like when blue was the winning color you avoided it, choosing the 50/50 chance of winning $5 at least once.

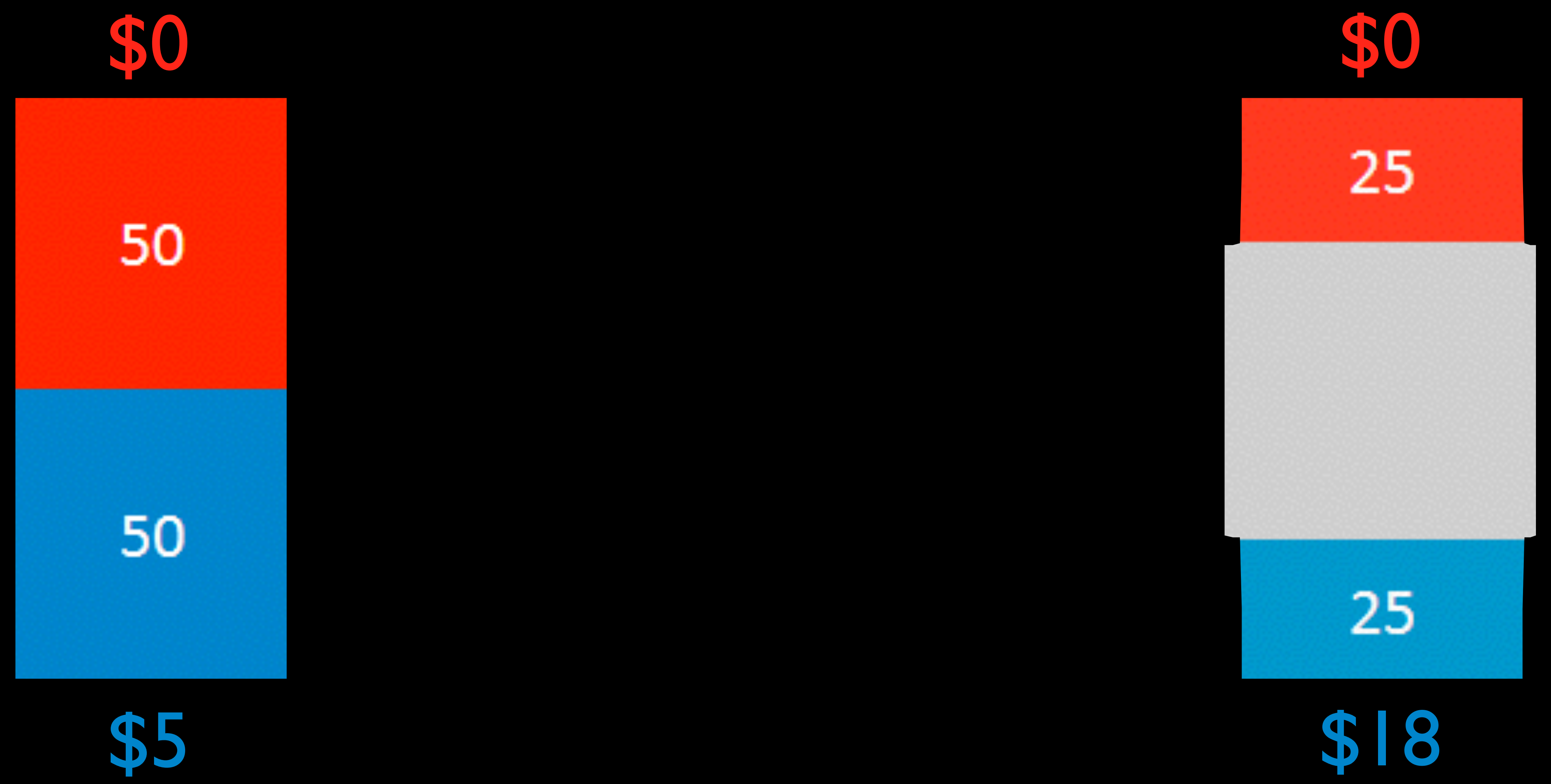

INCORRECT

Is it always the best choice to avoid these lotteries? Let's explore this question.

First, what is your best guess as to how many blue chips are in this lottery?

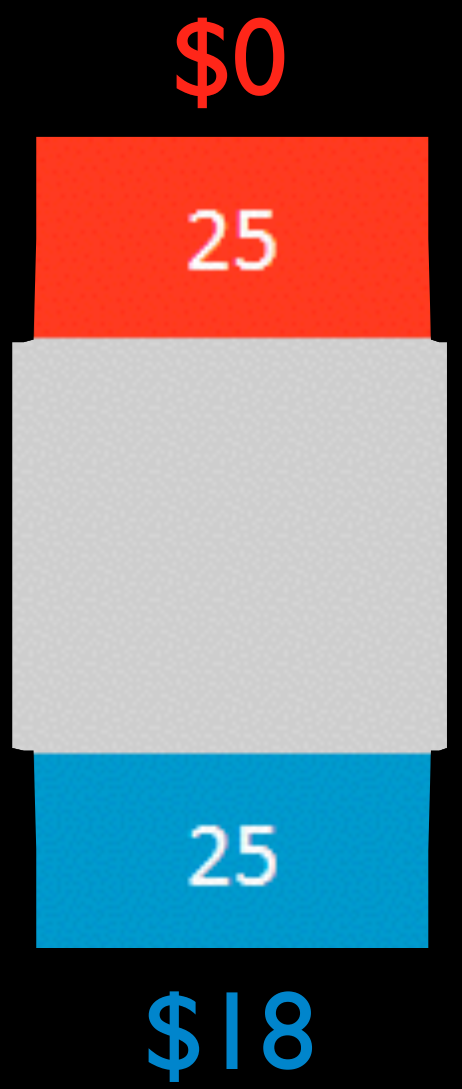

___25___out of 100

INCORRECT

If there are *25* blue chips in this lottery, how many red chips must there be? Remember, there are a total of 100 blue and red chips, so there are 100 - *25* red chips in the lottery.

____75____ red chips

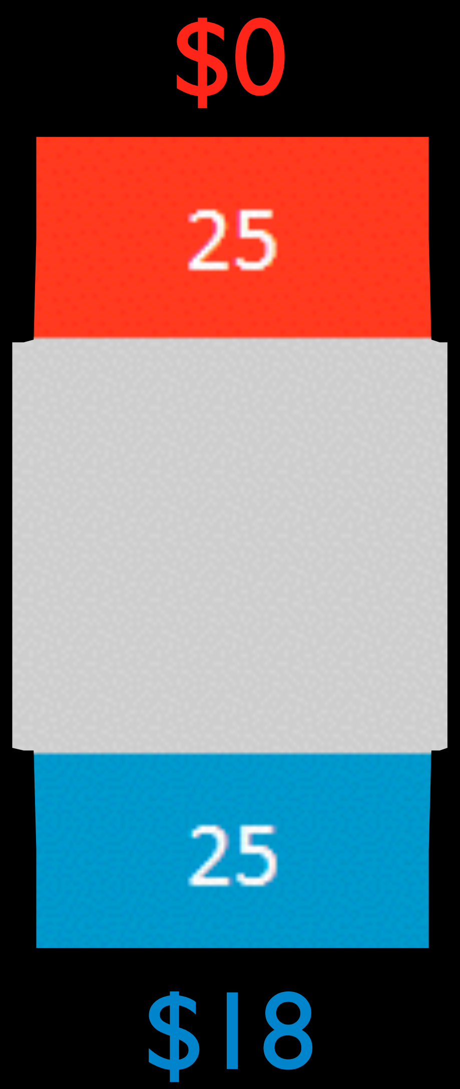

INCORRECT

Assuming there are 25 blue chips in this lottery and 75 red chips, what is your chance of winning (out of 100) when blue is the winning color?

25 out of 100

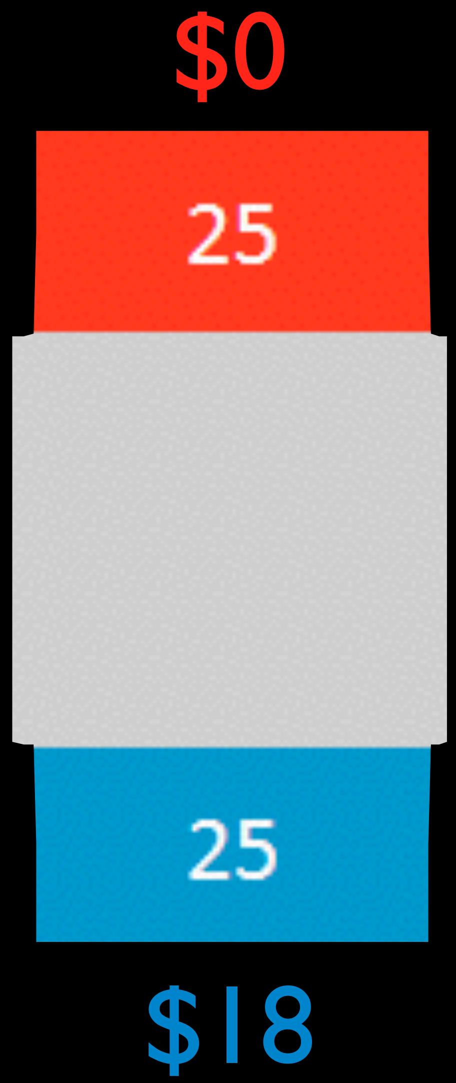

INCORRECT

Great! Now remember that each time you see this lottery the number of red and blue chips is the same, regardless of whether red or blue wins. Assuming that the numbers you chose are true, what is your chance of winning when red is the winning color?

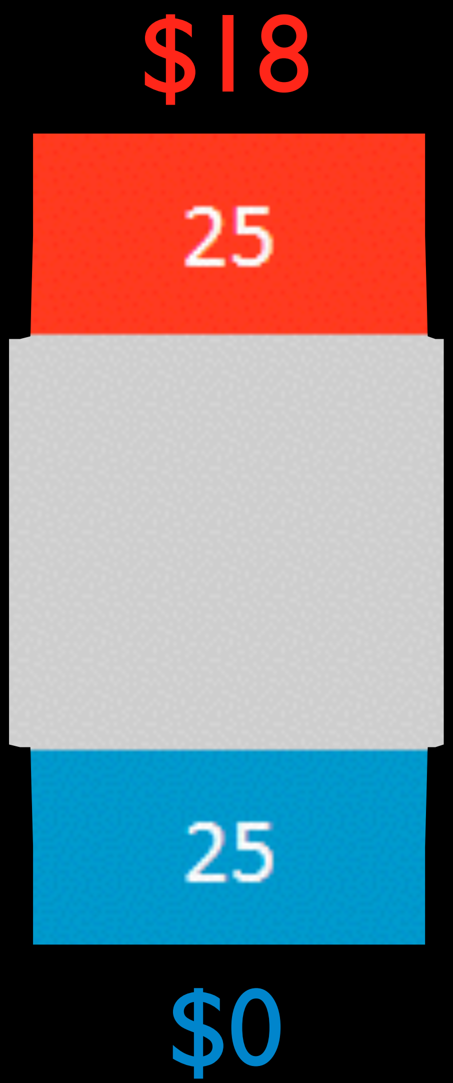

75 out of 100

Further, remember there are an equal number of lotteries with red as the winner as lotteries with blue as the winner. This means that there is an equal chance that a lottery with blue as the winner and with red as the winner will be played for real money at the end of the experiment.

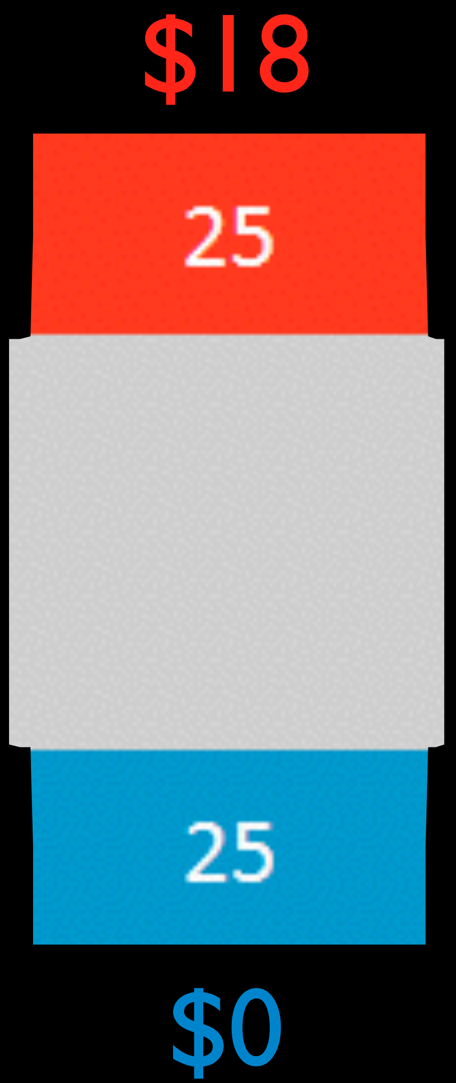

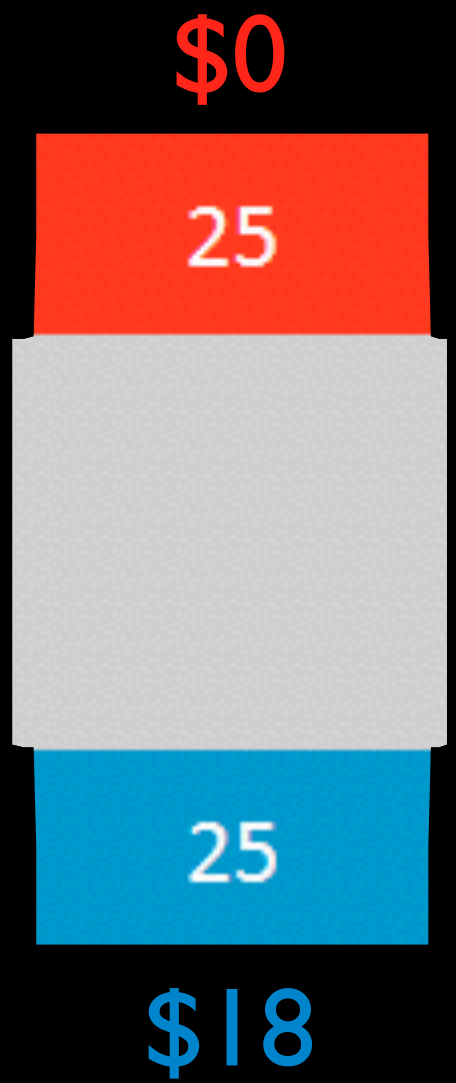

This means that your chance of winning is equal to the average of your chance of winning when blue is the winner (25 out of 100) and your chance of winning when red is the winner (75 out of 100).

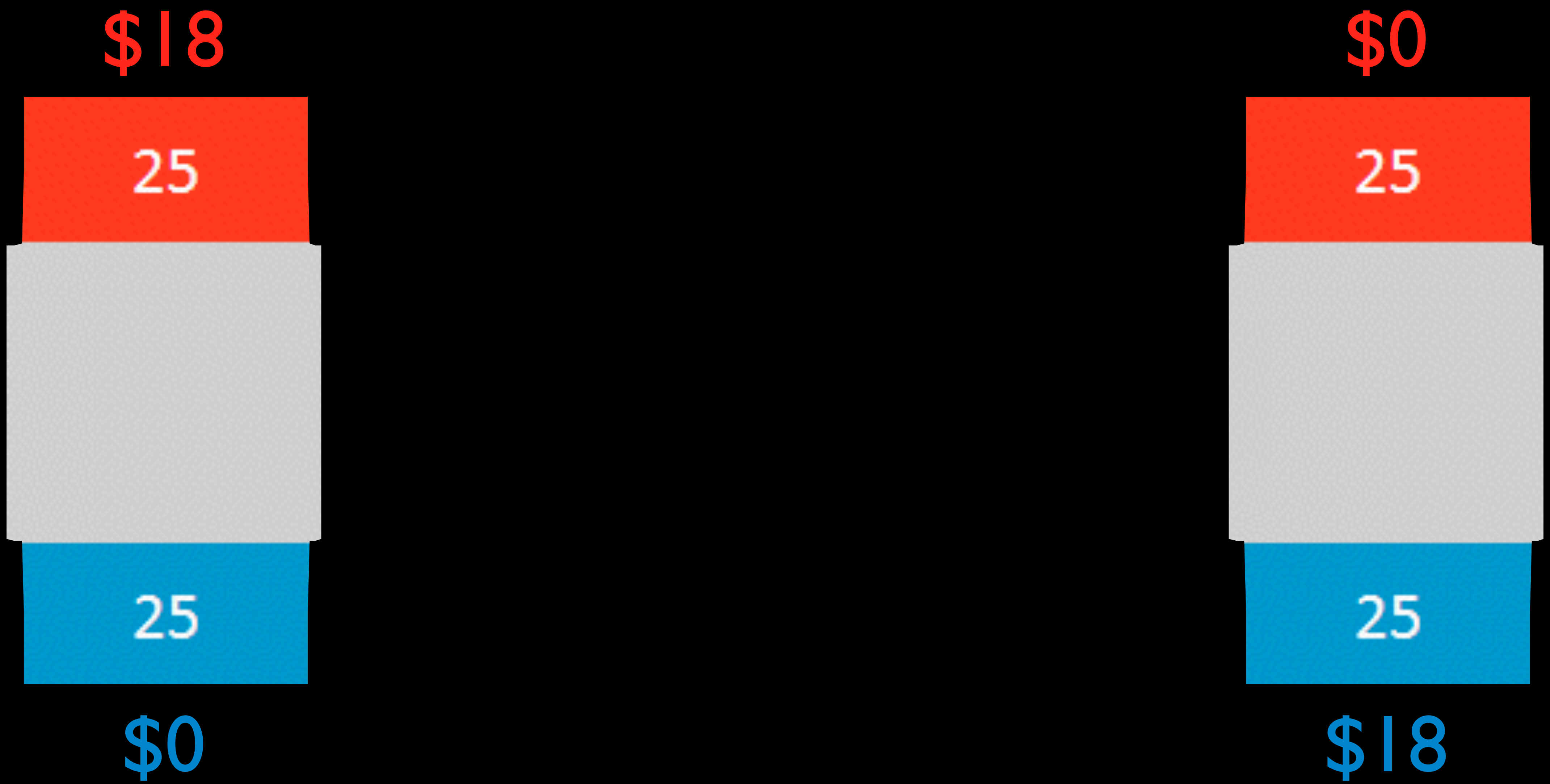

INCORRECT

What is your chance of winning across these two lotteries? (Reminder: you estimated your chance of winning when blue was the winner as *25* out of 100, and when red was the winner as *75* out of 100).

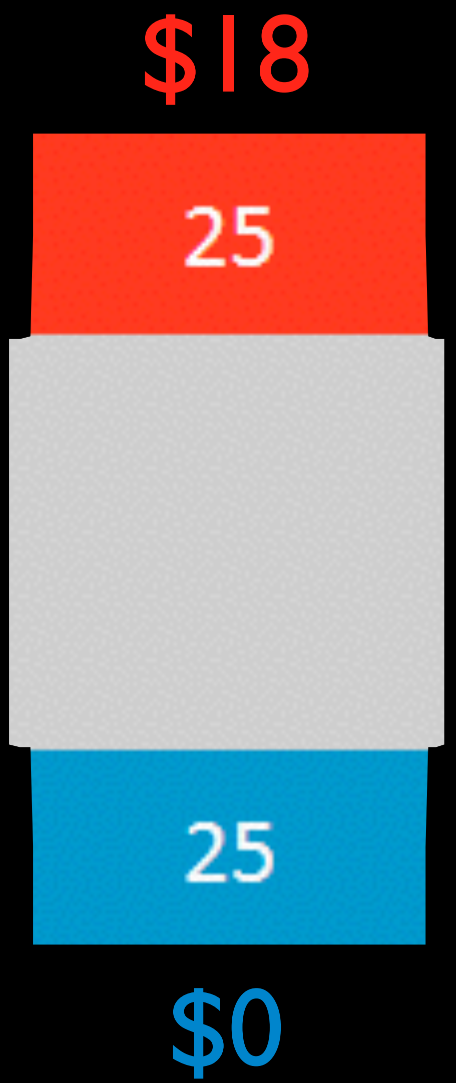

50 out of 100

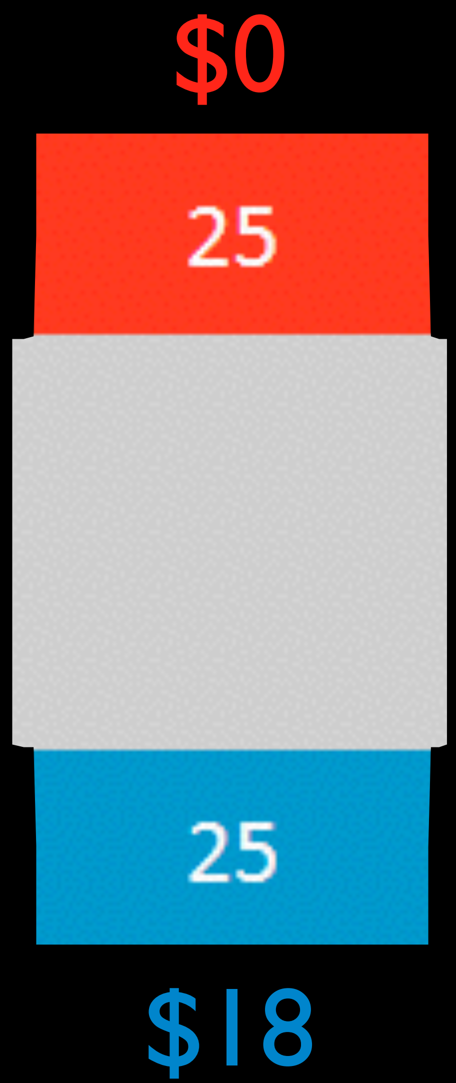

As you can see, your chance of winning in these lotteries is actually 50 out of 100. This is the exact same chance of winning as in the lottery paying only $5. Since your chance of winning in both lotteries is the same, it makes sense to choose the lottery that pays more

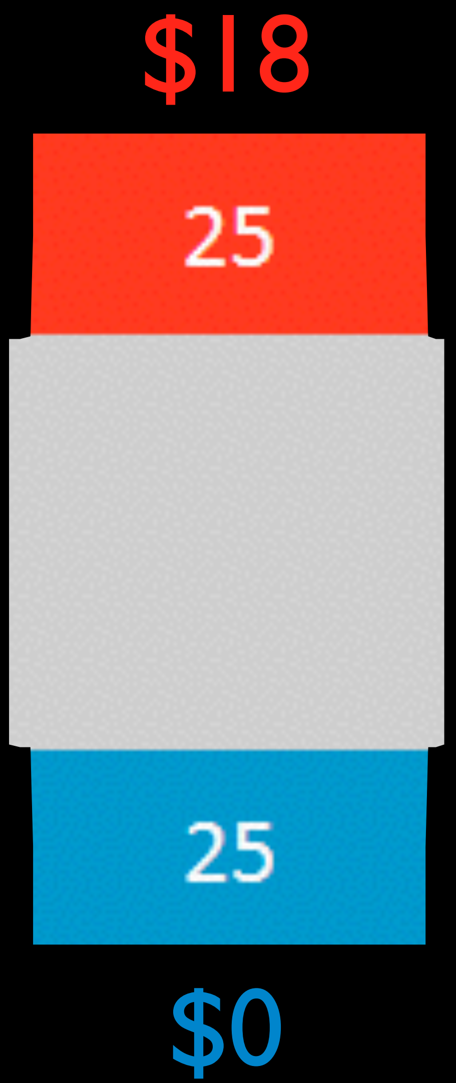

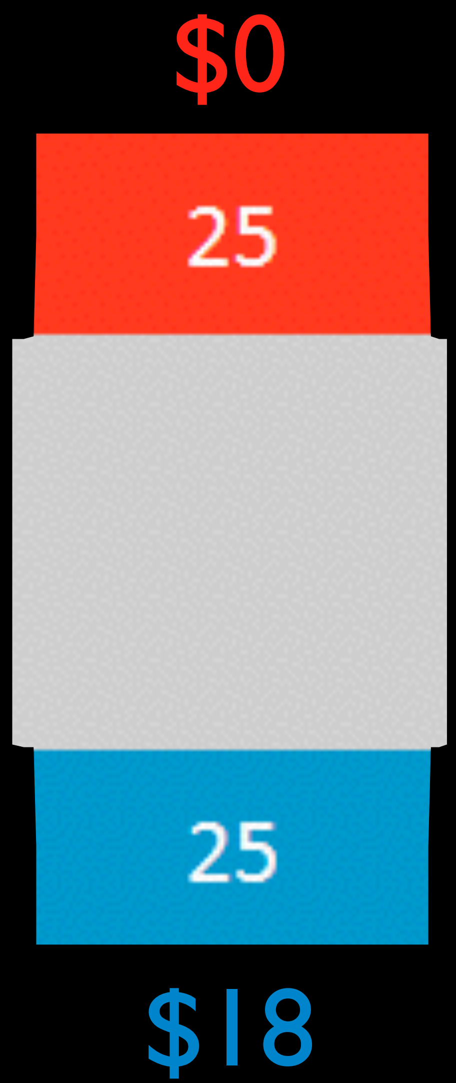

The fact that most people avoid these lotteries, even though they have an equal chance of winning more money, is a paradox in human decision-making termed ‘The Ellsberg Paradox’

FEEDBACK FOR INCORRECT RESPONSES

RETURN INCORRECT

Remember, there are at least 25 blue chips and at most 75 blue chips. Now, what is your guess as to how many blue chips are in this lottery?

25 out of 100

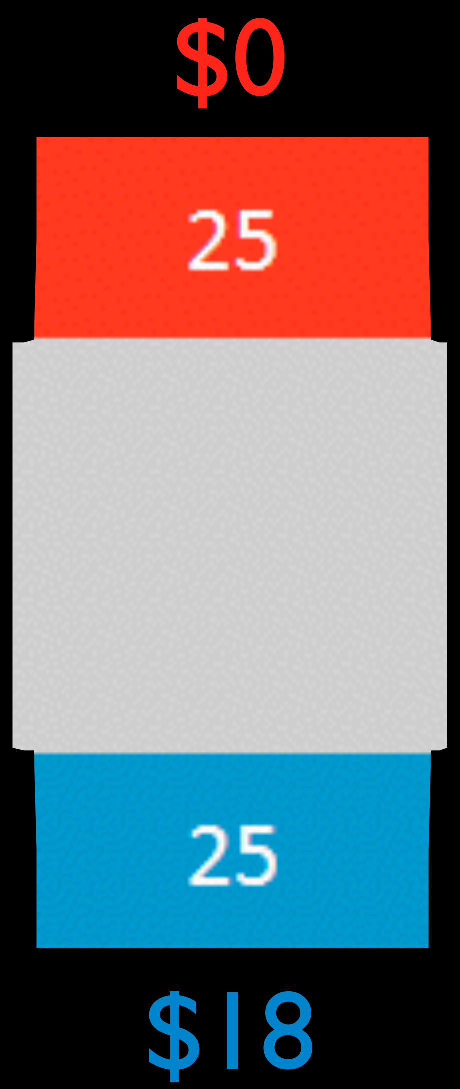

RETURN

Remember, there are at least 25 blue chips and at most 75 blue chips. Let’s assume the worst case scenario, that there are only 25 blue chips in this lottery.

25 out of 100

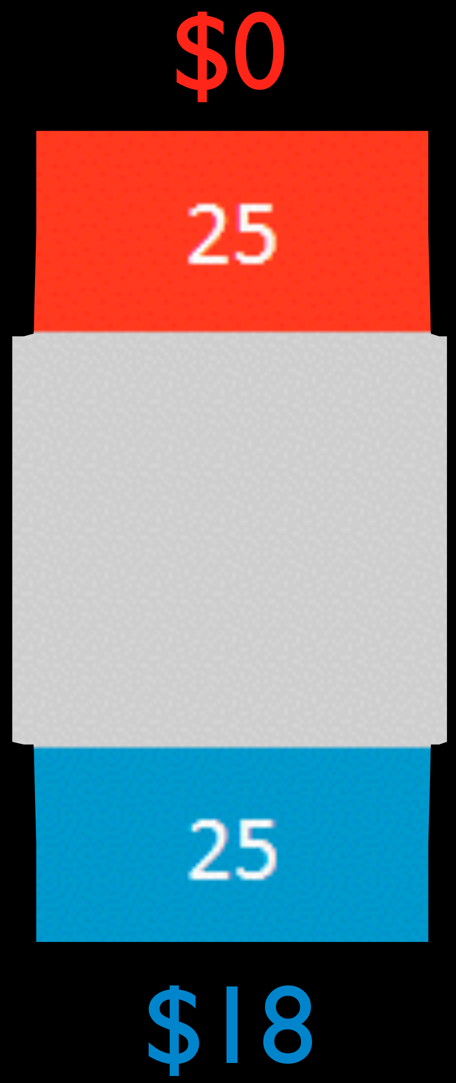

RETURN INCORRECT

Remember, if there are 25 blue chips in this lottery, there are 100 - 25 red chips in the lottery. How many red chips must there be in the lottery?

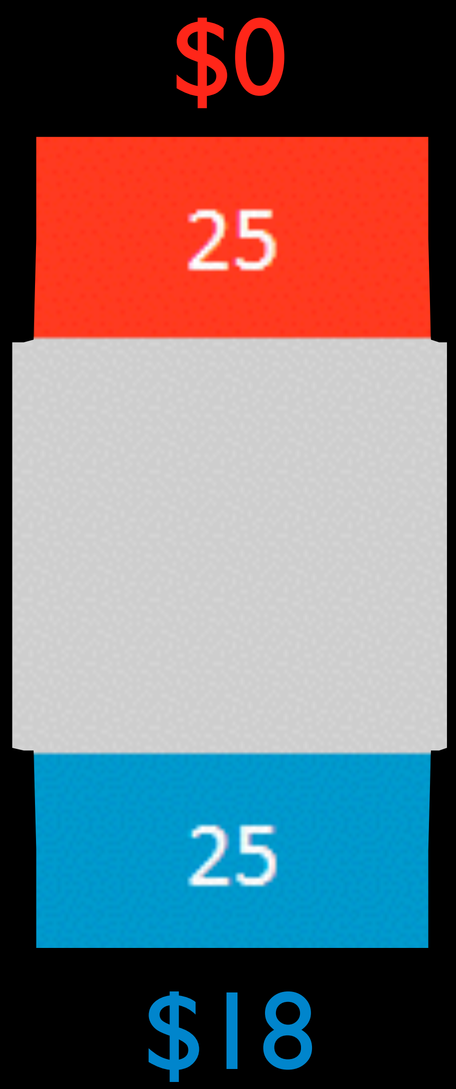

___75___ red chips

RETURN

Remember, if there are 25 blue chips in this lottery, there are 100 - 25 red chips in the lottery. That means there must be 75 red chips in the lottery.

____75___ red chips

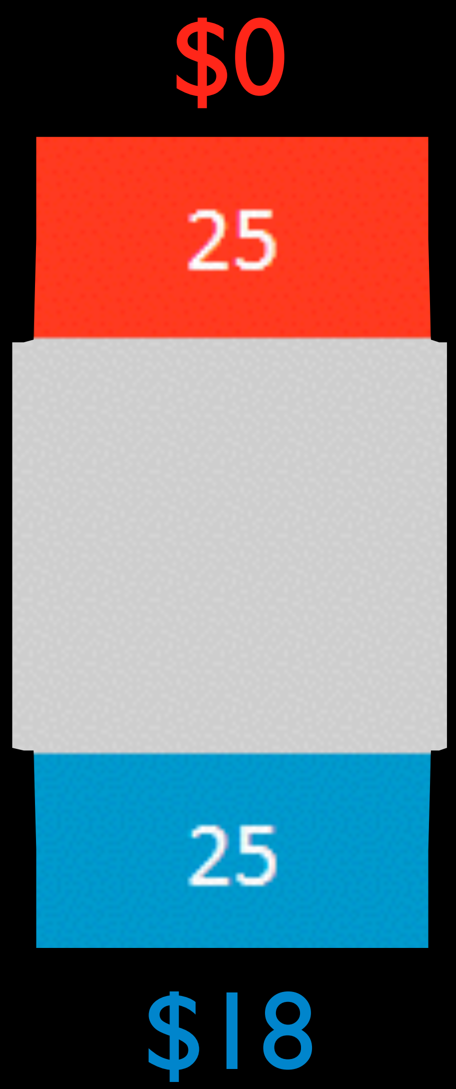

RETURN INCORRECT

Remember, there are 100 chips total. You think there are 25 blue chips in this lottery and 75 red chips. Assuming the numbers you chose are true, what is your chance (out of 100) of winning when blue is the winning color?

___25___ out of 100

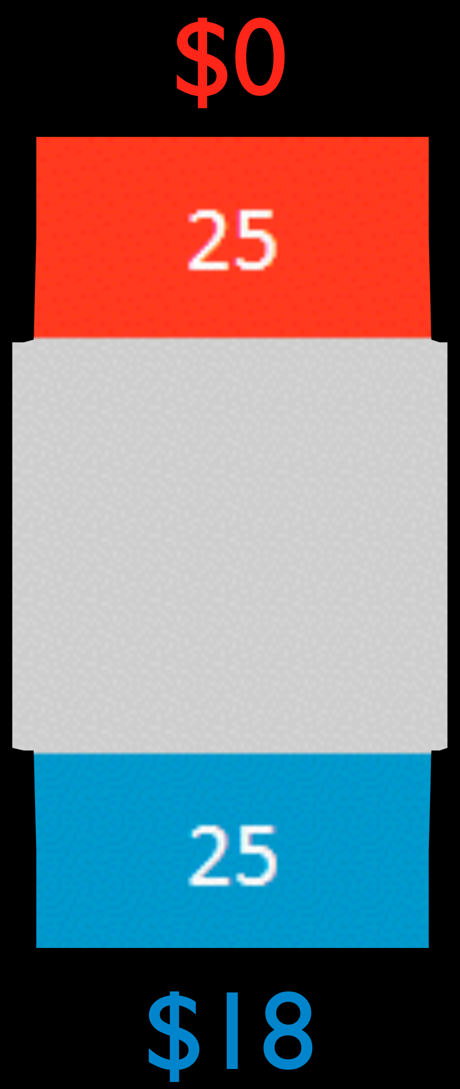

RETURN

Remember, there are 100 chips total. You think there are 25 blue chips in this lottery and 75 red chips. Assuming the numbers you chose are true, this means there is a 25 out of 100 chance that you will pick blue as the winning color.

25 out of 100

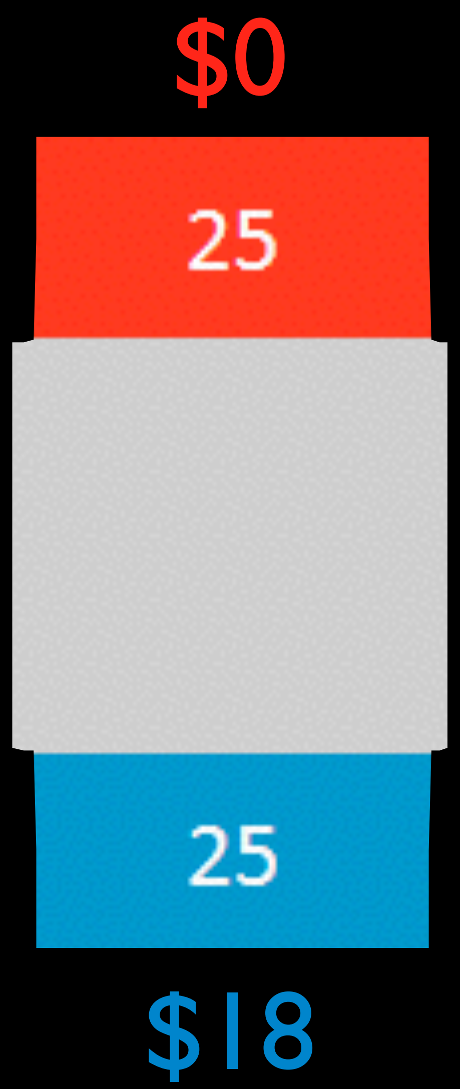

RETURN INCORRECT

Remember, there are 100 chips total. You think there are 25 blue chips in this lottery and 75 red chips.

Assuming the numbers you chose are true, what is your chance (out of 100) of winning when red is the winning color?

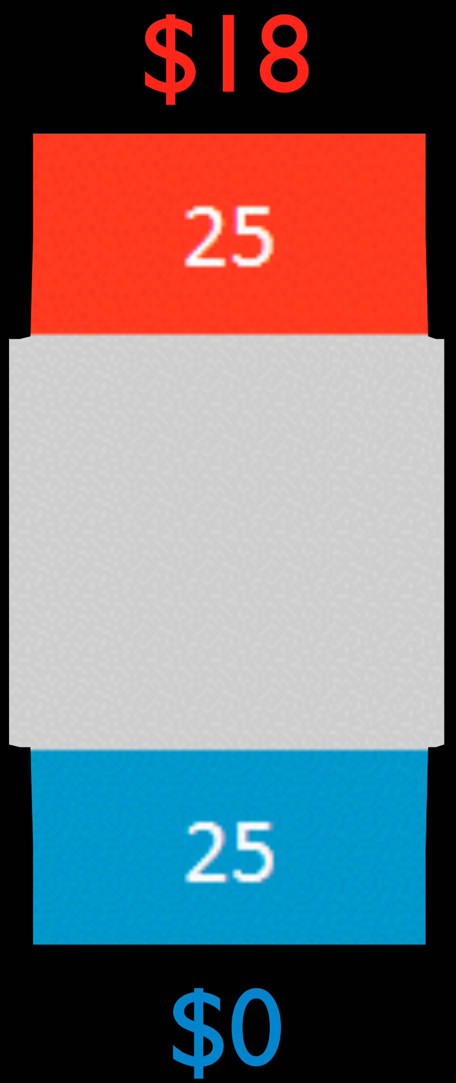

___75___ out of 100

RETURN

Remember, there are 100 chips total. You think there are 25 blue chips in this lottery and 75 red chips.
Assuming the numbers you chose are true, this means there is a 75 out of 100 chance that you will pick red as the winning color.

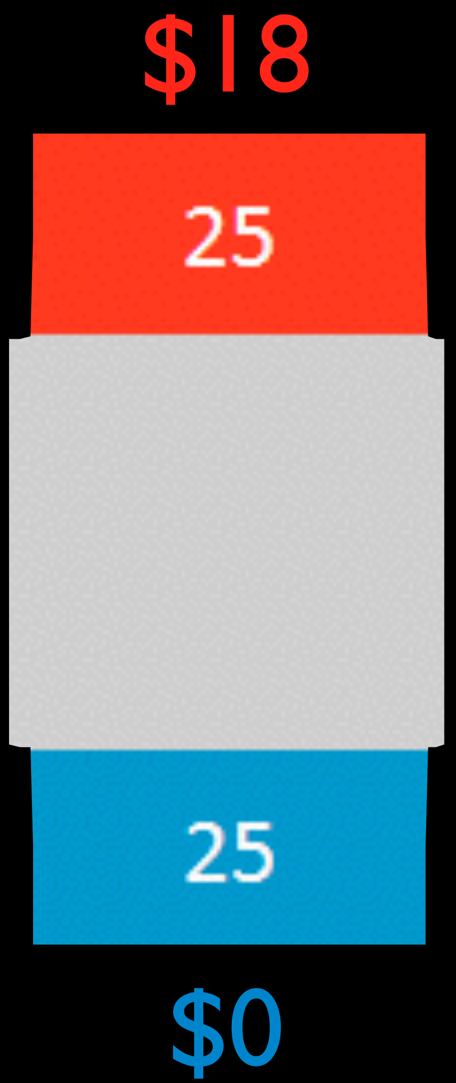

___75___ out of 100

RETURN

Remember, you estimated your chance of winning when blue was the winner as 25 out of 100, and when red was the winner as 75 out of 100. This means that your chance of winning is (25 + 75) / (100 + 100). This is equal to 50 out of 100.

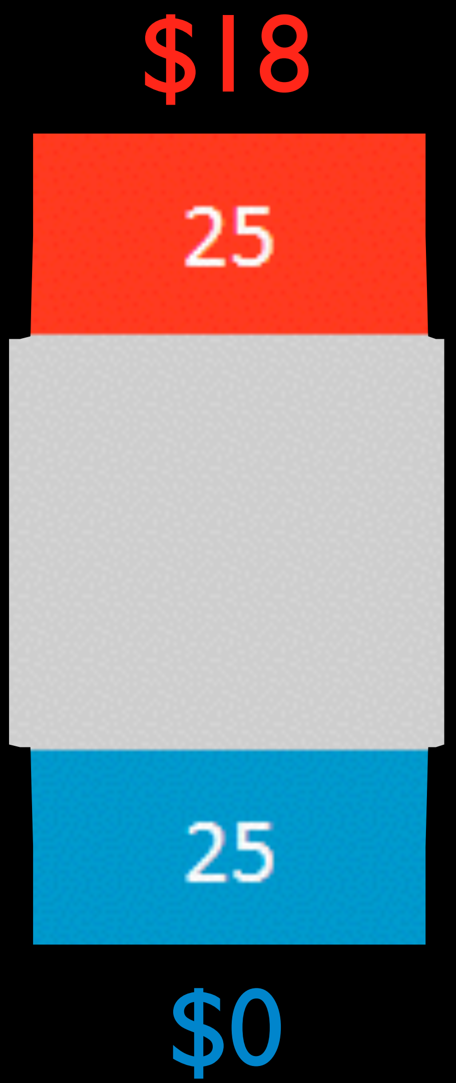

___*50*___ out of 100

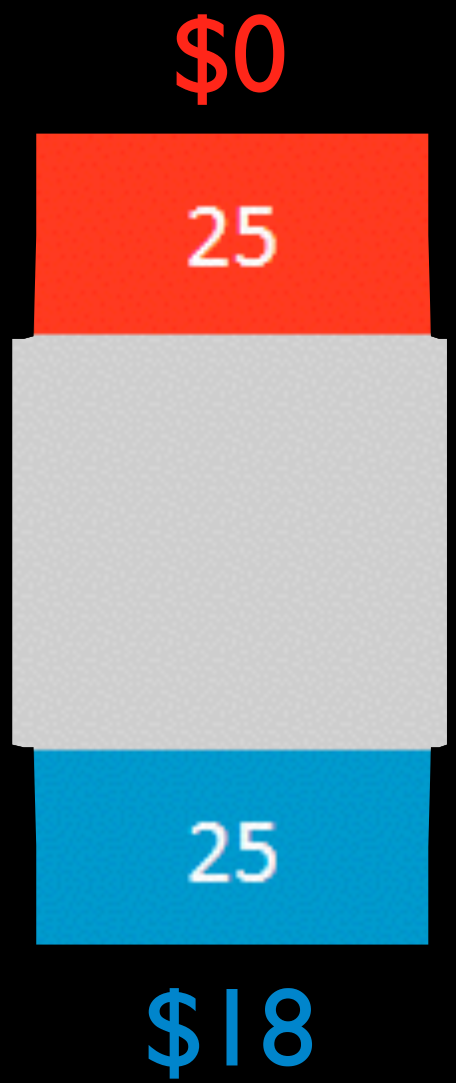

Non-active Calculation Condition

**Note: For this demonstration, text written in gray depends on responses and may be slightly different between participants. Also, the ambiguous lottery used for calculation is an example, and the actual one shown to the participant depends on her own choice in pre-intervention trials.*

In this experiment you have seen two types of lotteries. Those in which you know your exact chance of winning (like in the lottery on the left), and those in which you do not (like in the lottery on the right).

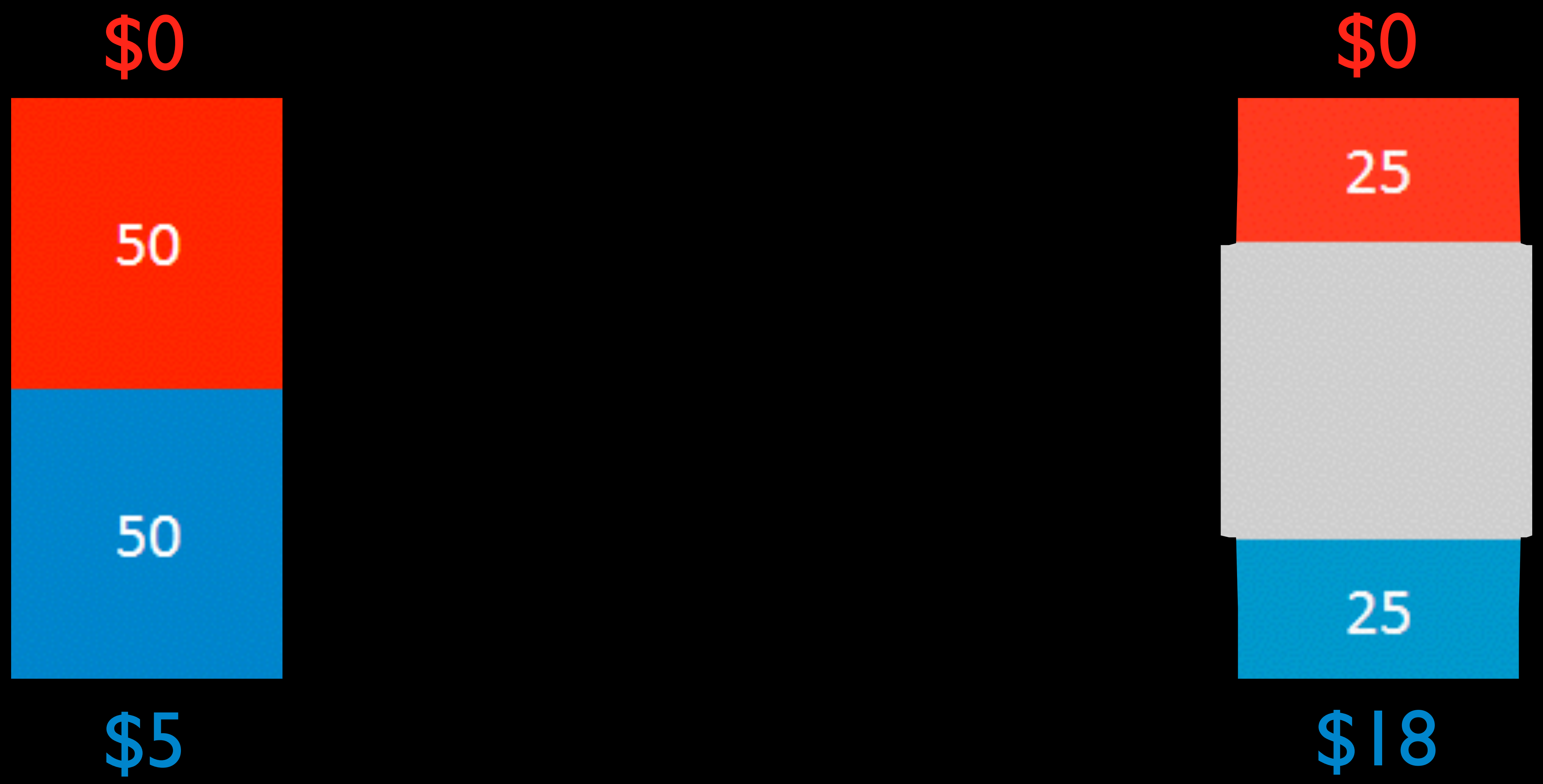

When people know their exact chance of winning different people make different choices. Some prefer taking low risks for small gains (like on the left) while others prefer taking high risks for large gains (like on the right). Choices on these kinds of lotteries are a matter of personal preference.

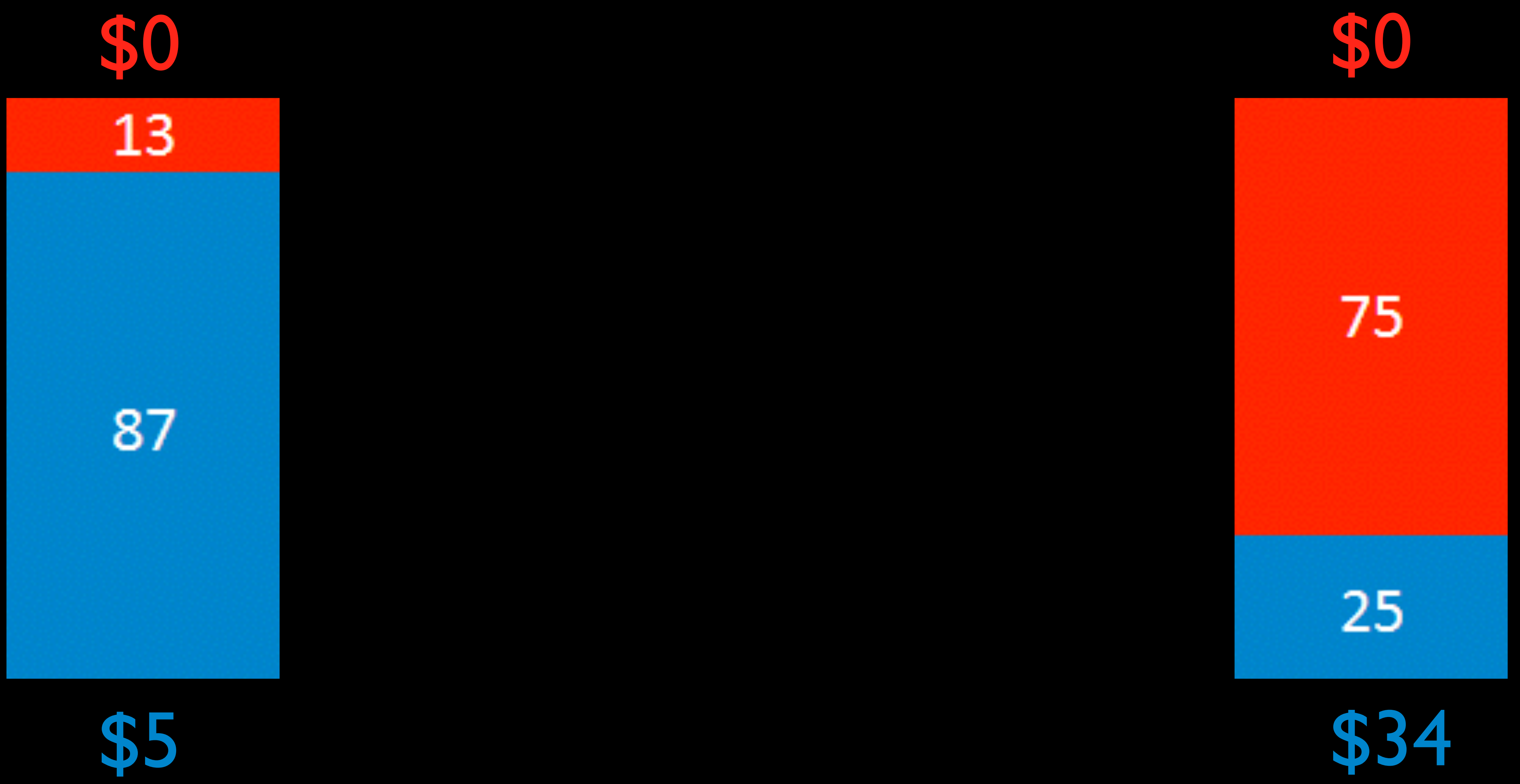

On the other hand, most people avoid playing lotteries when they don't know their exact chance of winning, like in the lottery below. In fact, when you were presented with this lottery, you avoided playing it, choosing the 50% chance of winning $5 at least once.

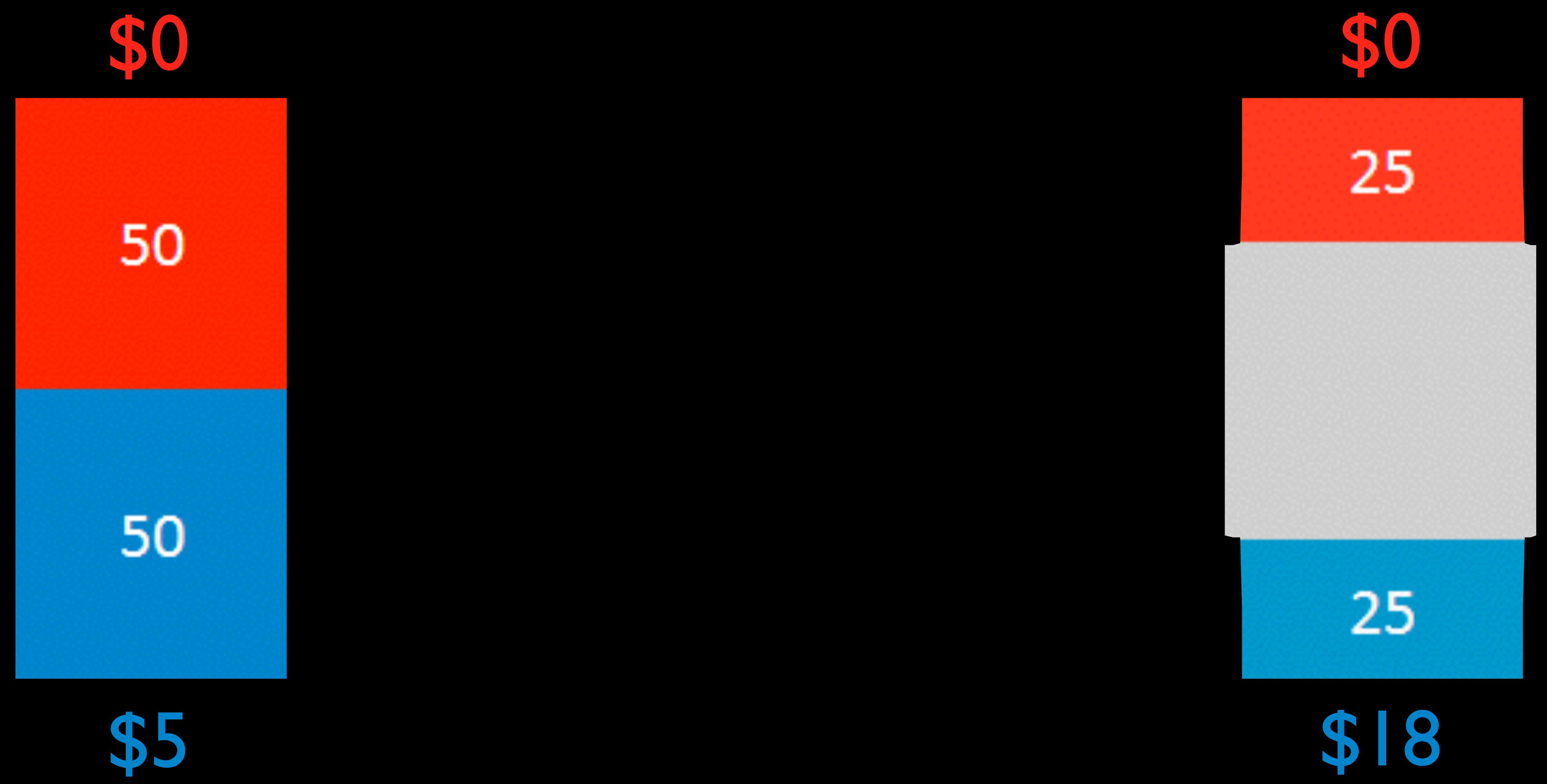

You were also presented with the same lottery, except the winning color was red instead of blue. Just like when blue was the winning color you avoided it, choosing the 50/50 chance of winning $5 at least once.

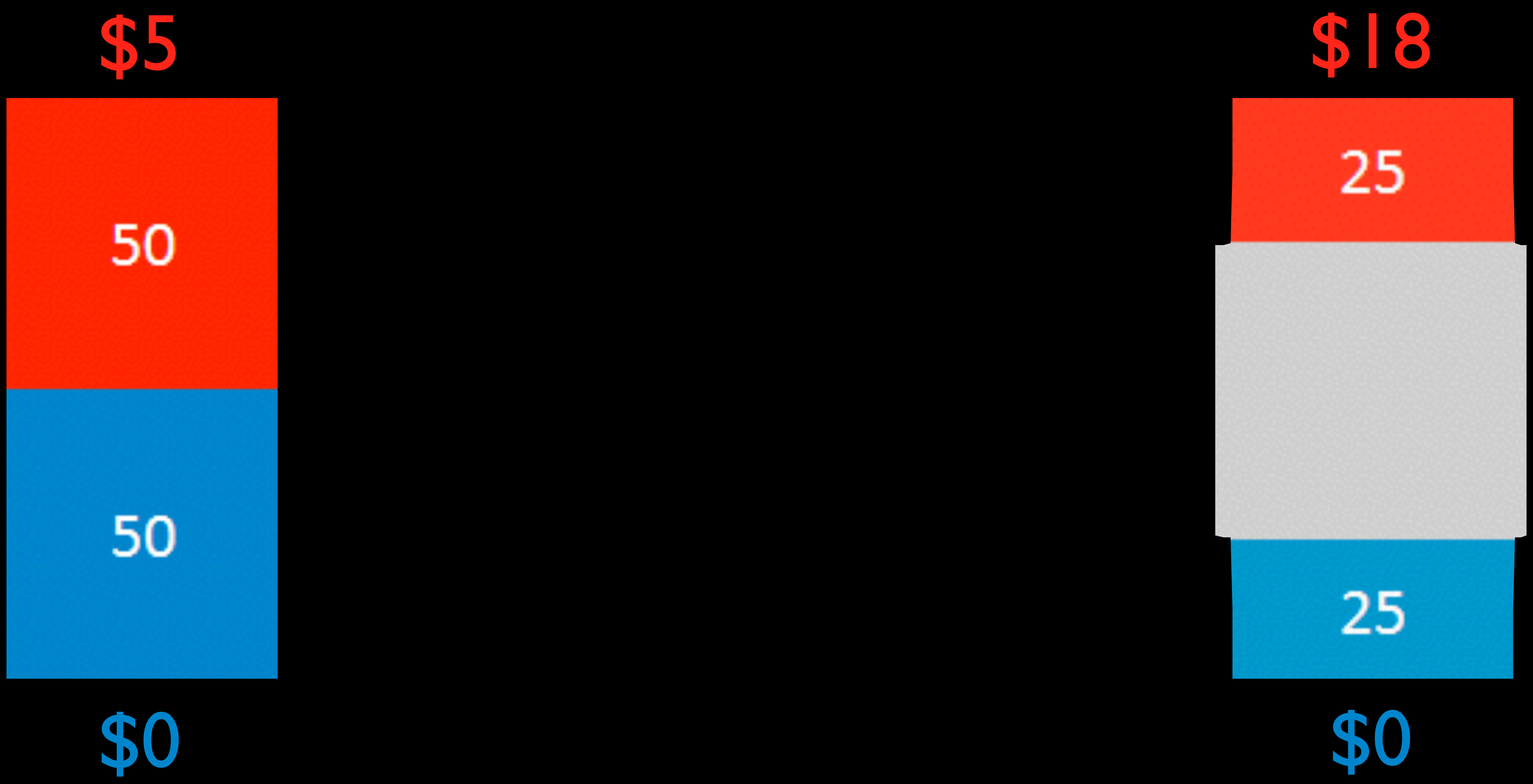

Is it always the best choice to avoid these lotteries? Let’s explore this question.

Let’s imagine the worst case scenario, that there are only 25 blue chips in the lottery.

25 out of 100

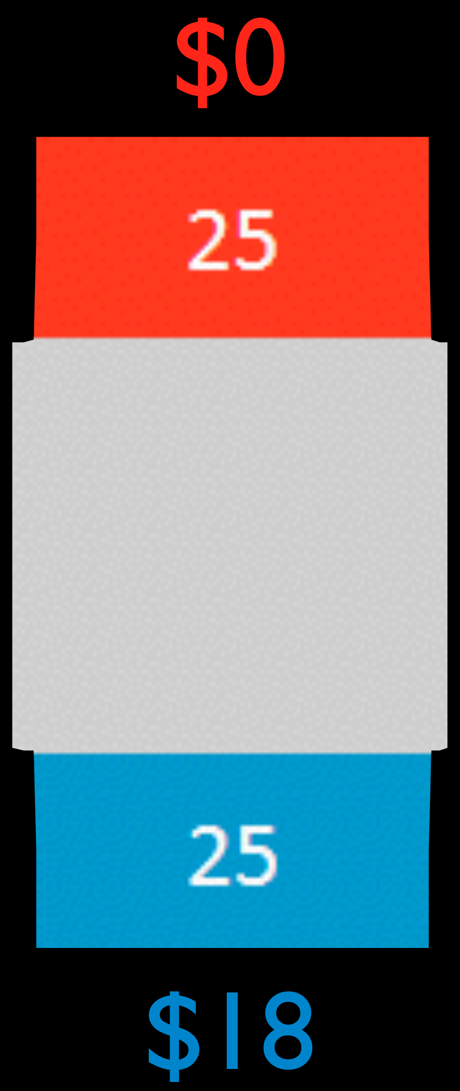

If there are 25 blue chips in this lottery, that means there must be 100 - 25 or 75 red chips, since there are a total of 100 blue and red chips.

75 red chips

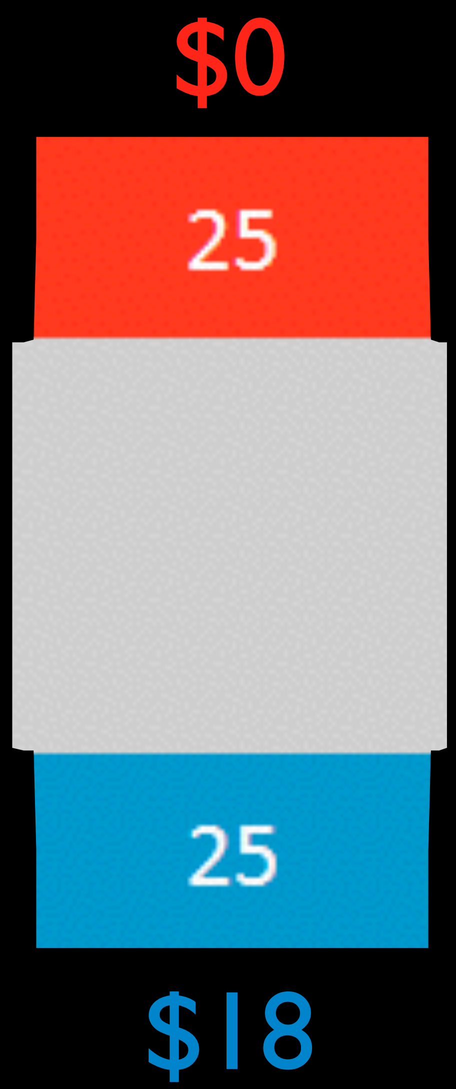

Assuming there are 25 blue chips in this lottery and 75 red chips, your chance of winning is 25 out of 100 when blue is the winning color.

25 out of 100

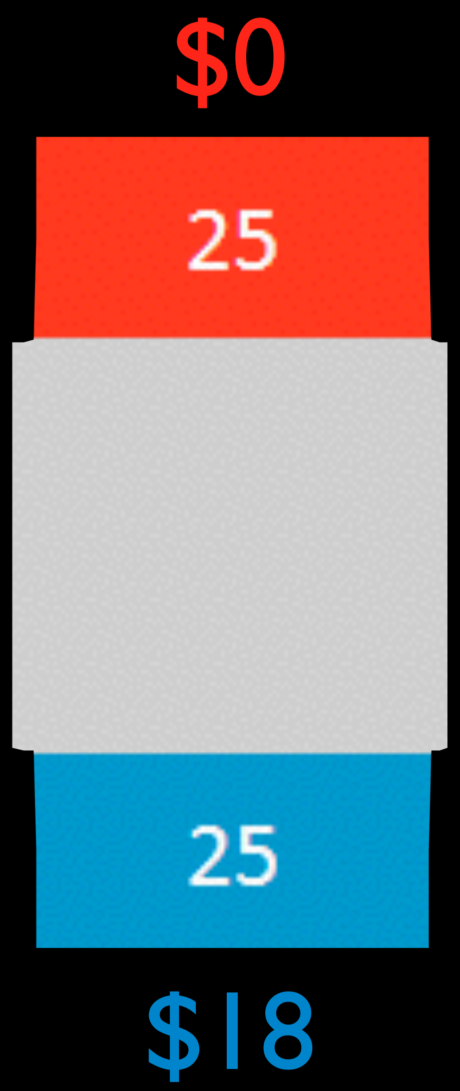

Now remember that each time you see this lottery the number of red and blue chips is the same, regardless of whether red or blue wins. Assuming that the numbers you chose are true, your chance of winning when red is the winning color is 75 out of 100.

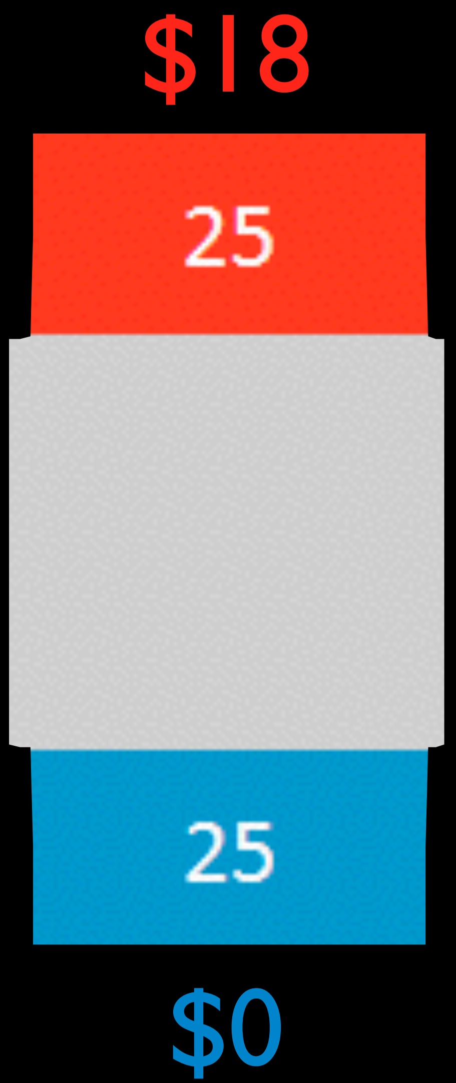

75 out of 100

Further, remember there are an equal number of lotteries with red as the winner as lotteries with blue as the winner. This means that there is an equal chance that a lottery with blue as the winner and with red as the winner will be played for real money at the end of the experiment.

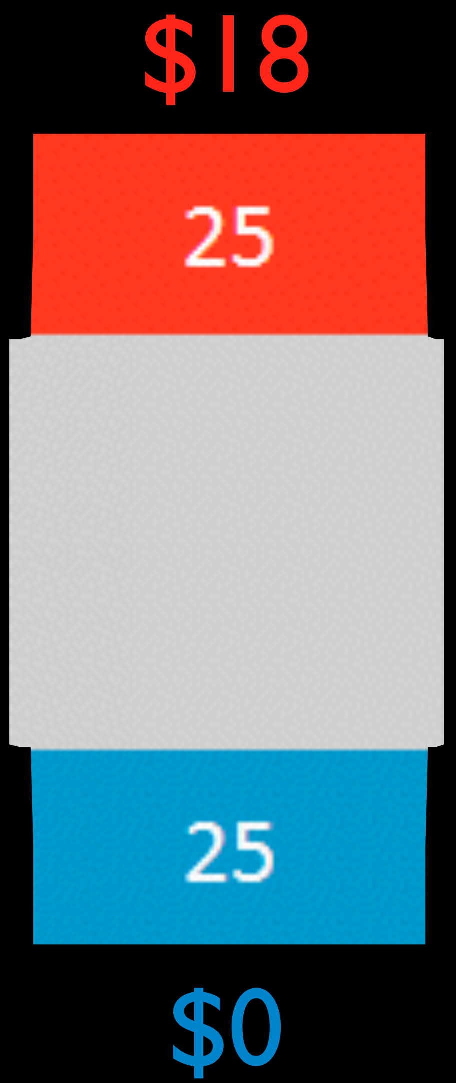

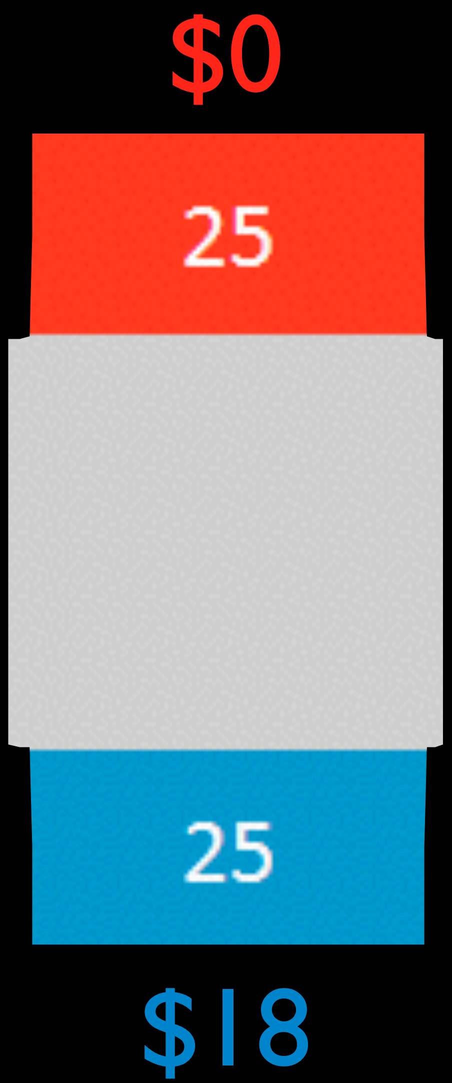

This means that your chance of winning is equal to the average of your chance of winning when blue is the winner (25 out of 100) and your chance of winning when red is the winner (75 out of 100).

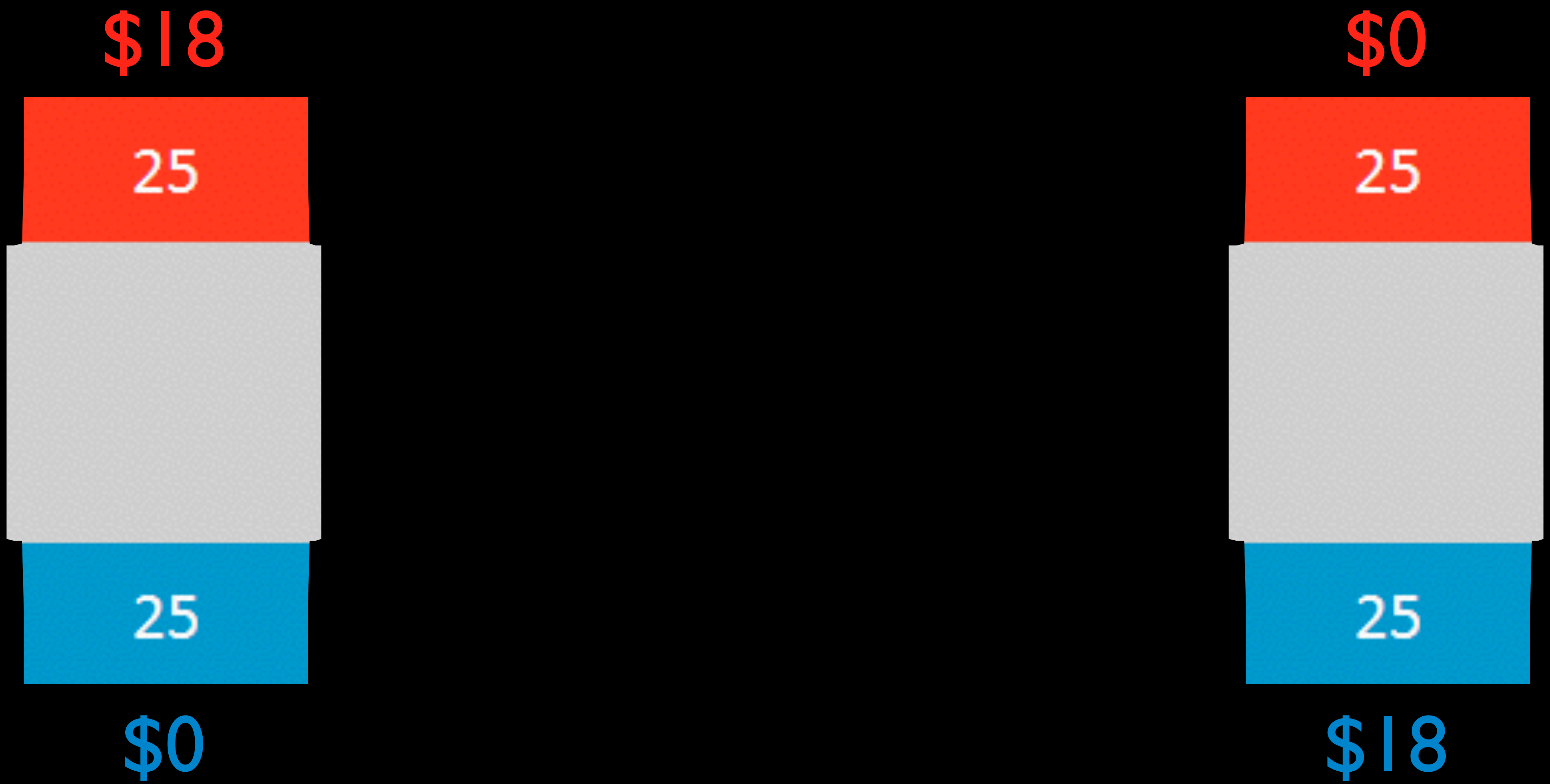

Therefore, your chance of winning across these two lotteries is equal to the average of your chance of winning when blue was the winner (25 out of 100) and when red was the winner (75 out of 100).

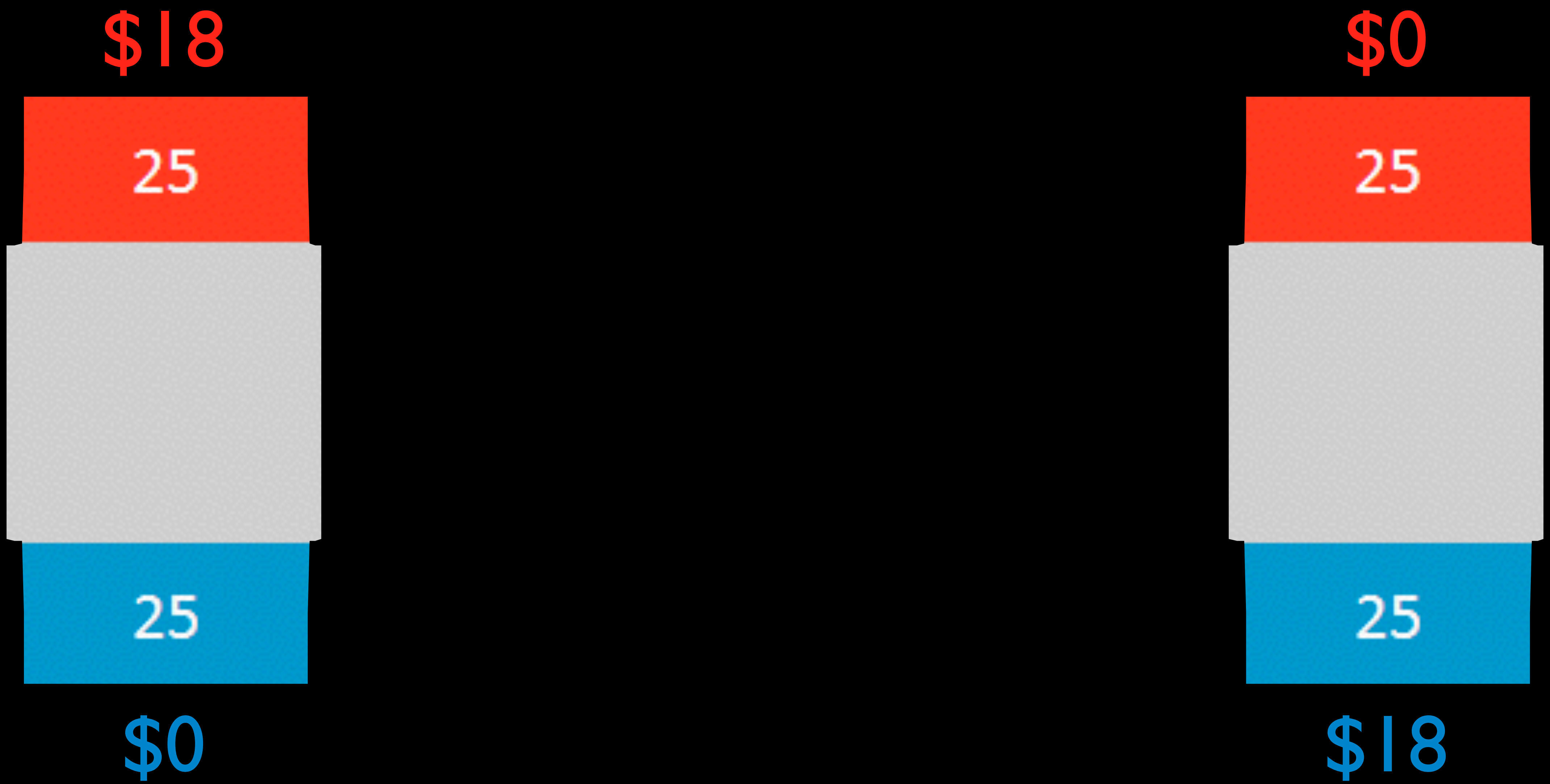

Your chance of winning in these lotteries is actually 50 out of 100 because (25 + 75)/2 = 50. This is the exact same chance of winning as in the lottery paying only $5. Since your chance of winning in both lotteries is the same, it makes sense to choose the lottery that pays more.

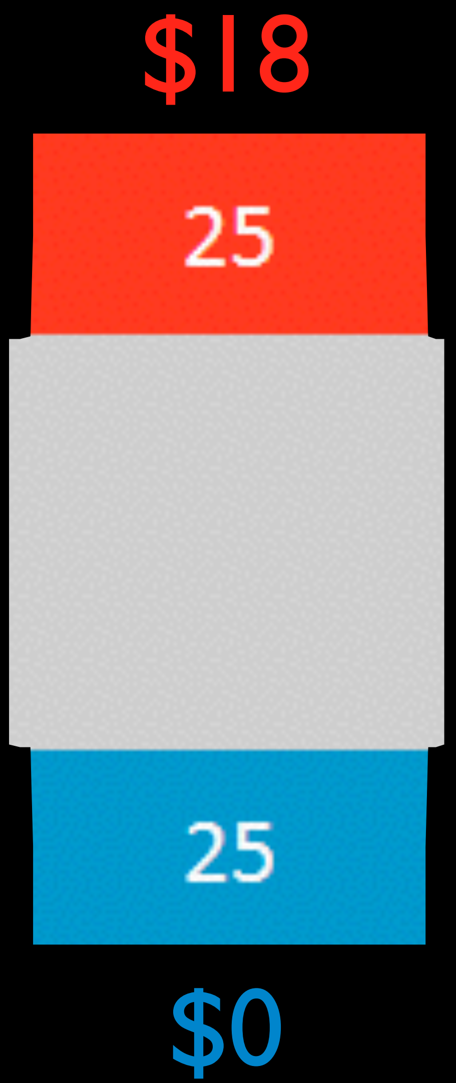

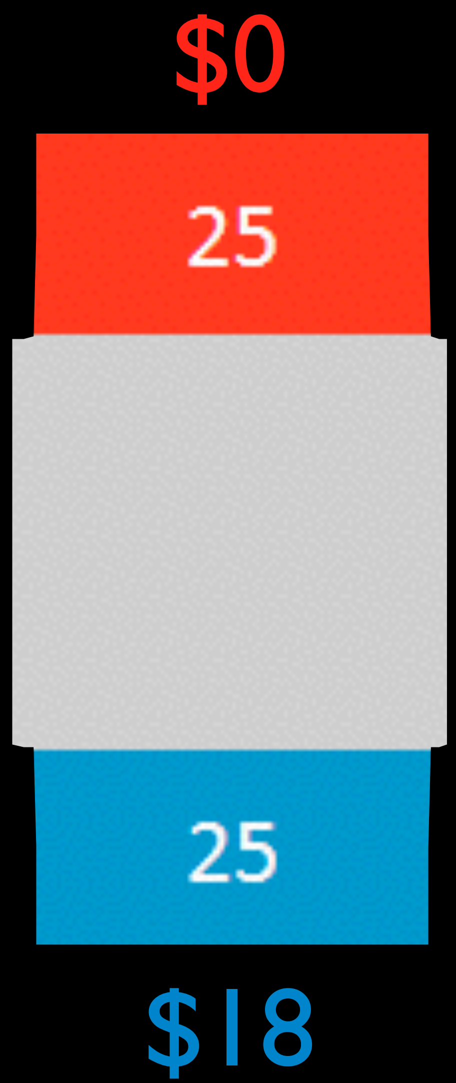

The fact that most people avoid these lotteries, even though they have an equal chance of winning more money, is a paradox in human decision-making termed ‘The Ellsberg Paradox’

Base Rate Condition

**Note: For this demonstration, text written in gray depends on responses and may be slightly different between participants. Further,* INCORRECT *buttons have been added to demonstrate feedback for errors.*

One of the tools psychologists use to study decision-making is something called the Taxicab problem, which goes something like this.

(press “Enter” to advance)

Imagine a cab was involved in a hit and run accident at night. There are 100 total cabs in the city. 15 of them are blue and 85 are green.

A witness identified the cab as blue.

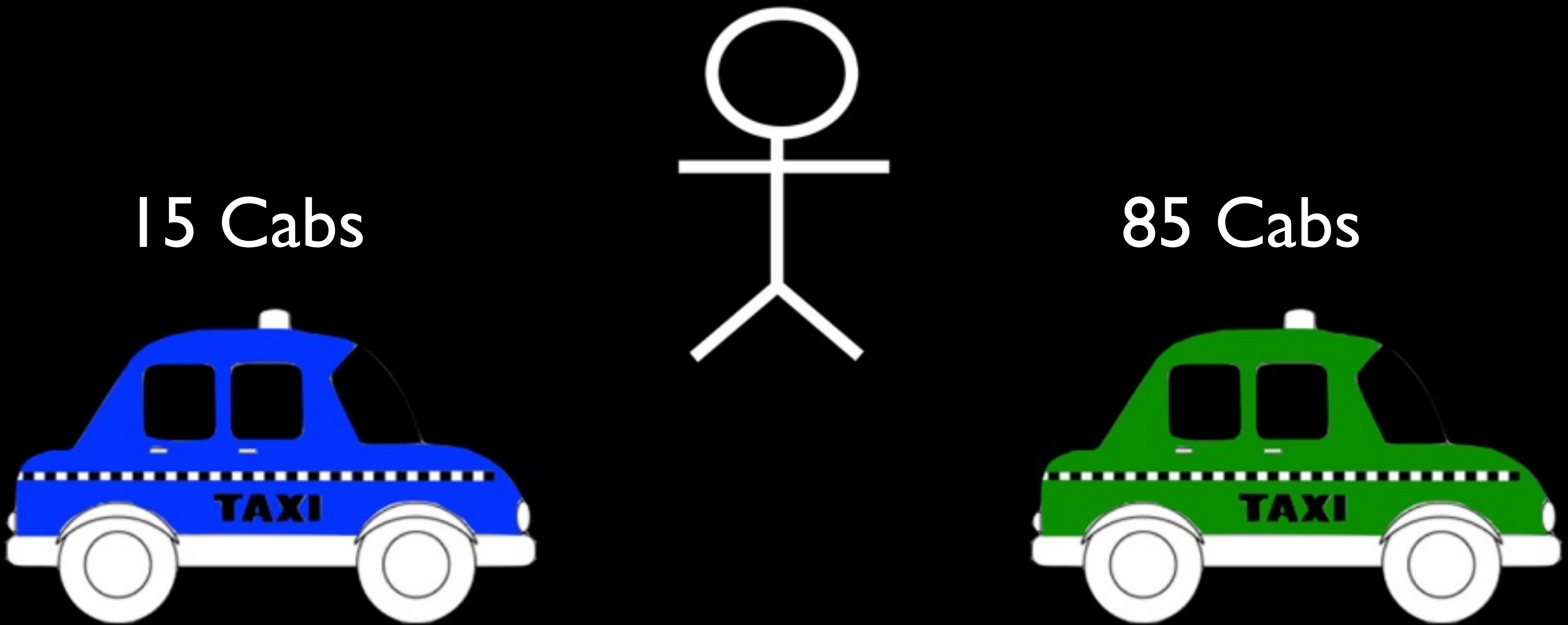

To test the reliability of the witness, the court showed him 100 green and blue cabs under the same circumstances as on the night of the accident. The witness correctly identified the color for 80 of the cabs, and incorrectly identified the color for 20 of the cabs.

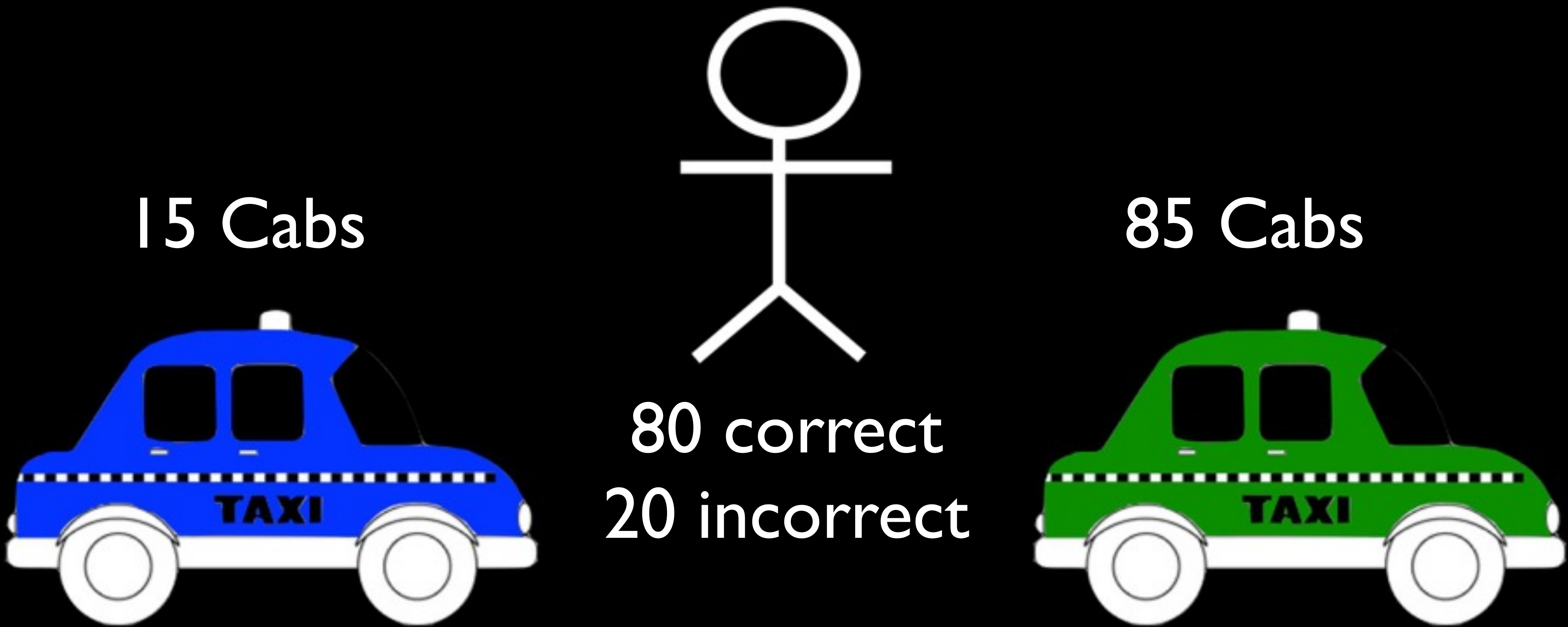

INCORRECT

Given that the witness identified the cab involved in the accident was blue, what do you think the chance is that it was blue rather than green?

out of 100

15 Cabs

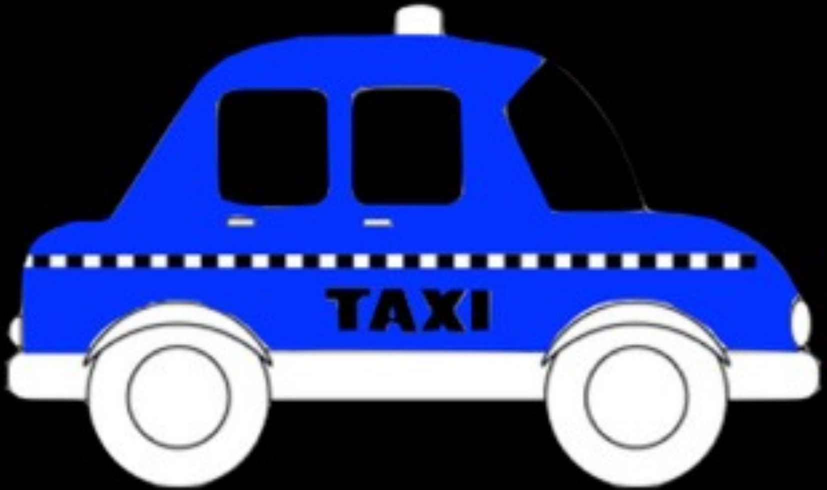

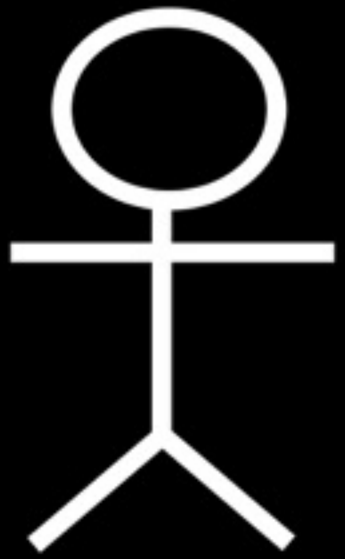

80 correct
20 incorrect

85 Cabs

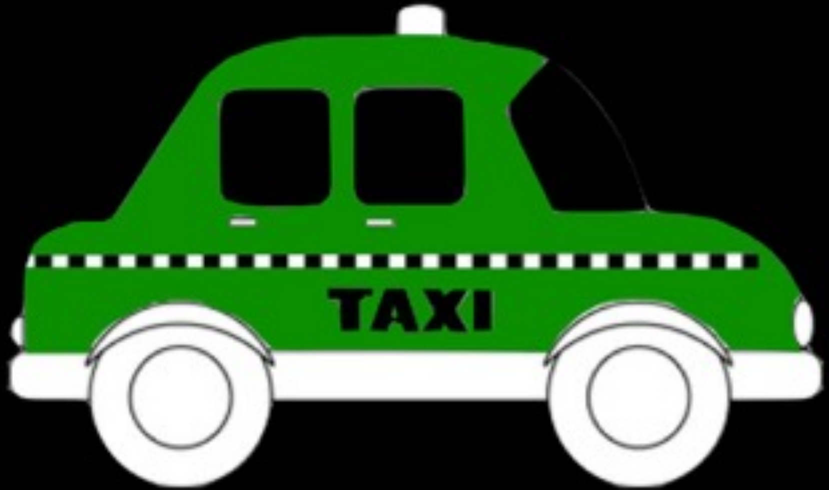

Like you, most people guess the chance that the cab was blue to be over 50 out of 100, generally estimating the chance the cab was blue to be about 80 out of 100. However, the chance the cab actually was blue is actually much smaller than that!

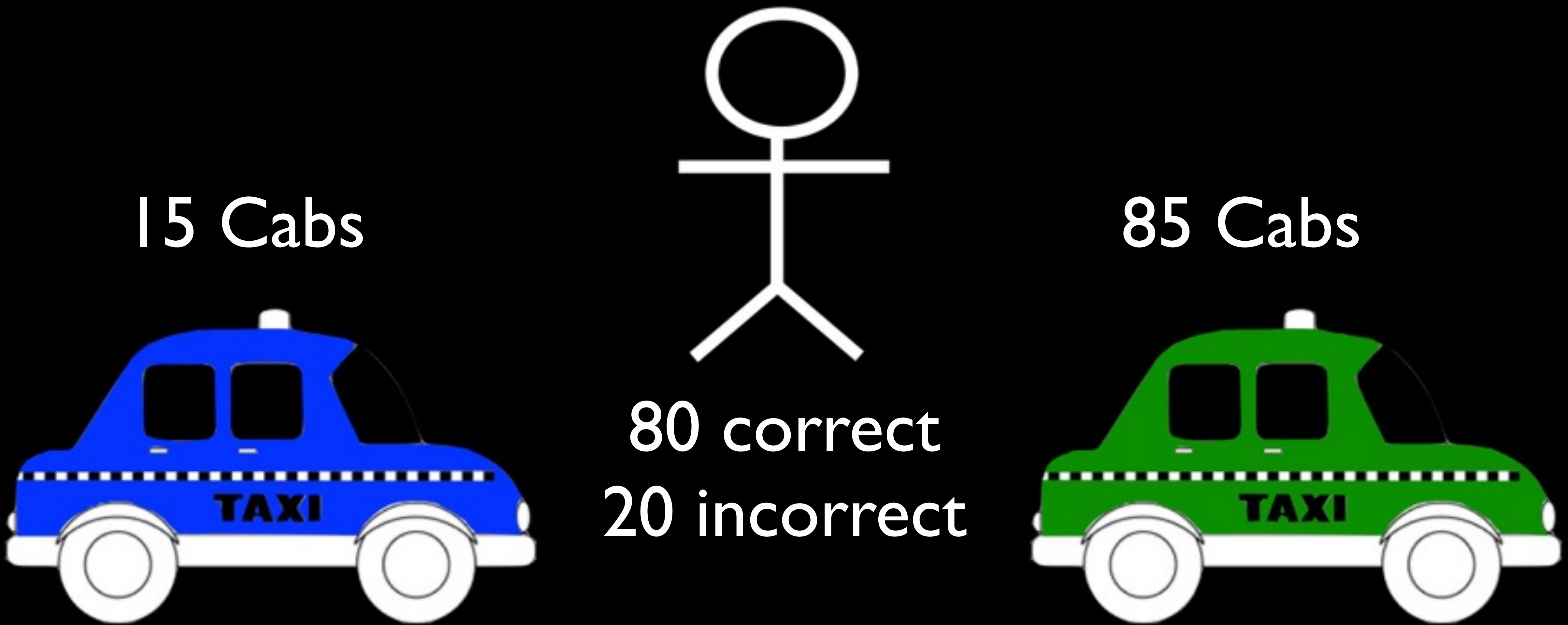

To determine the chance the witness correctly identified the cab as blue, we need to consider not only the chance the witness correctly identified the cab as blue, but also the chance that the witness would identify any cab as blue, regardless of what color it actually is.

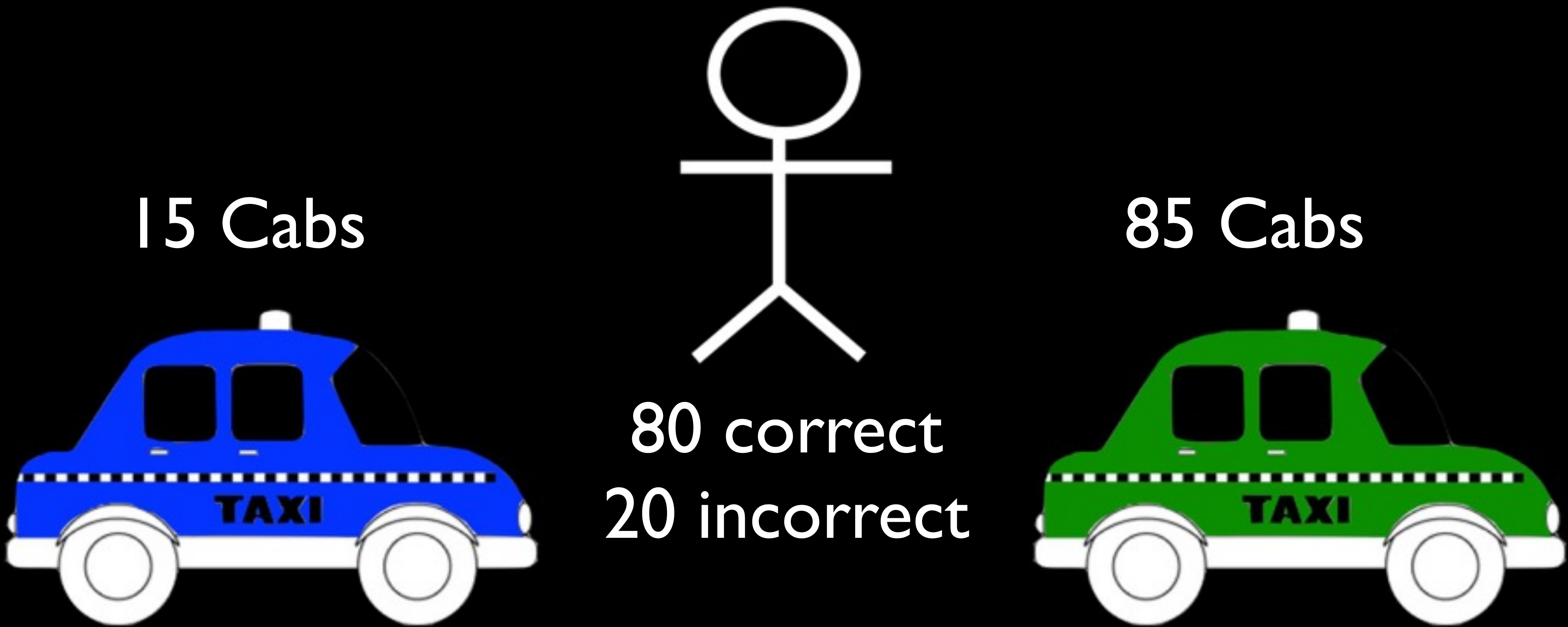

Therefore, in order to determine the chance that the cab involved in the accident was really blue, we need to know three things: the chance the witness correctly identified a blue cab as blue, the chance that the witness incorrectly identified a green cab as blue, and from these two values we can determine the chance that the witness would identify any cab as blue, regardless of the color.

15 Cabs

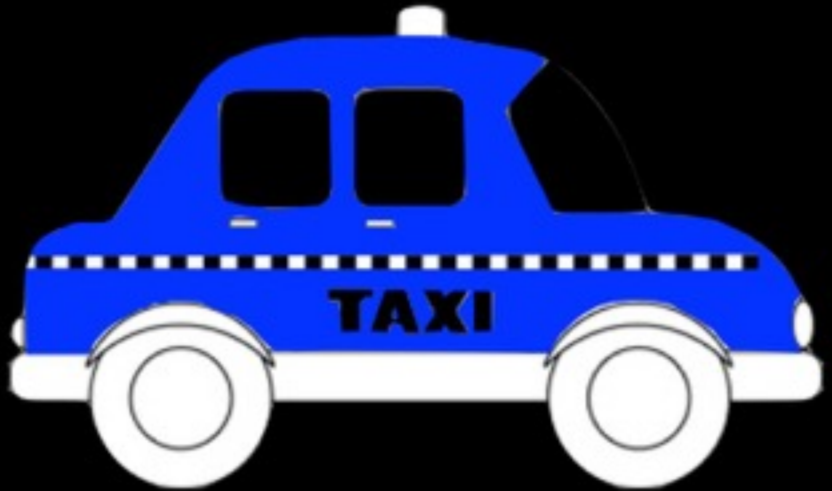

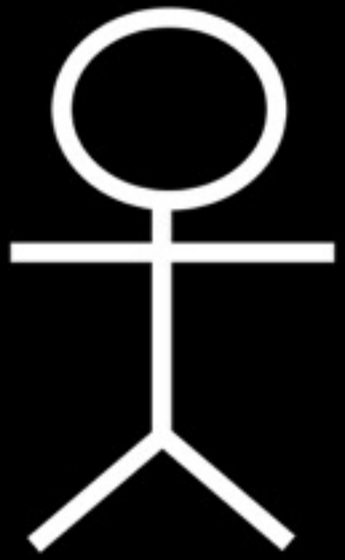

80 correct
20 incorrect

85 Cabs

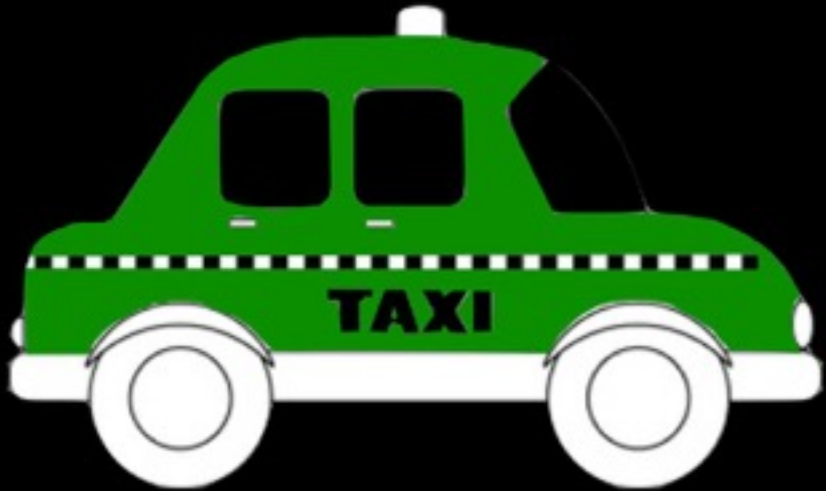

INCORRECT

First, what is the chance the witness correctly identified the blue cab as blue?

If you need a math refresher, keep in mind that the chance the witness correctly identified the blue cab as blue is equal to the chance that the cab was blue (15 out of 100) times the chance the witness was correct (80 out of 100).

out of 100

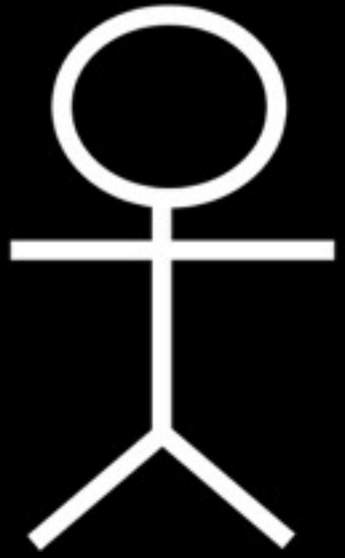

15 Cabs

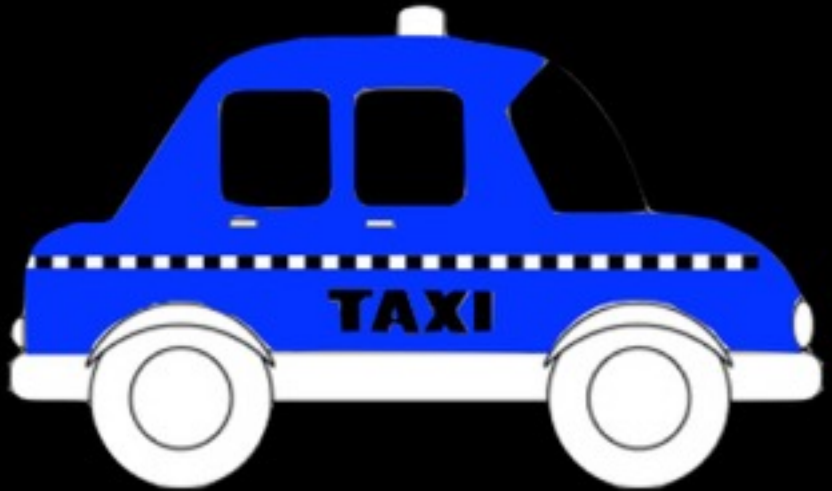

80 correct
20 incorrect

85 Cabs

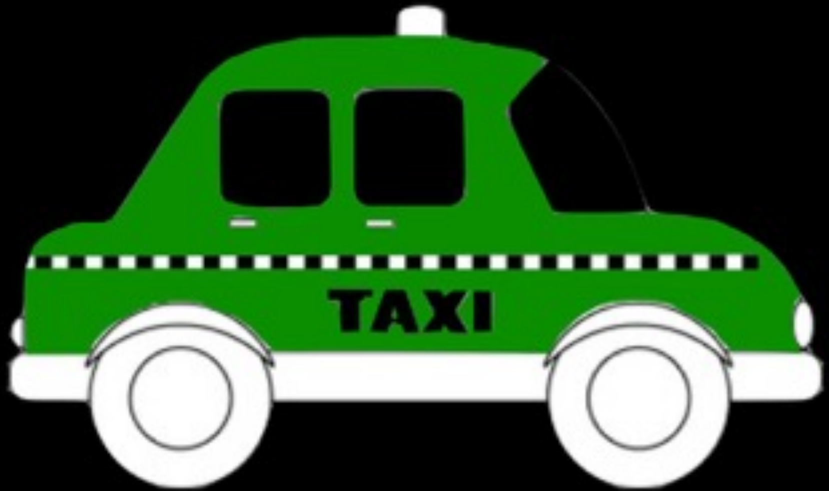

INCORRECT

Great! The chance the witness correctly identified the cab as blue is 12 out of 100. Our next step is to determine the chance the witness incorrectly identified a green cab as blue. What is the chance the witness incorrectly identified a green cab as blue?

Remember, 85 of the cabs in the city are green and the witness was wrong on 20 out of 100 attempts to identify the cab color. Therefore, there is an 85/100 times 20/100 chance the witness incorrectly identified a green cab as blue.

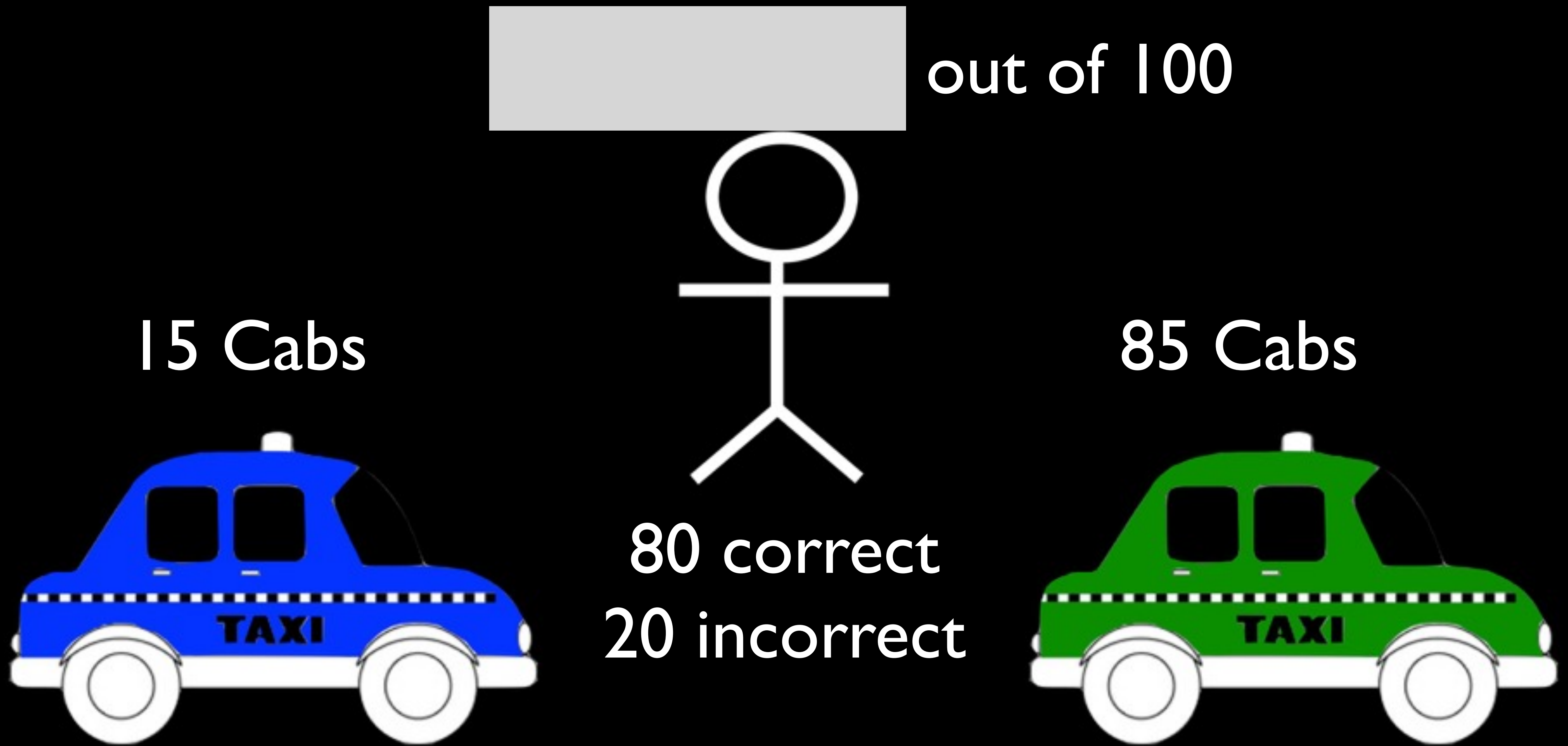

INCORRECT

Great! Last we need to determine the chance that the witness would identify ANY cab as blue, regardless of the color.
Remember, there is a 12 out of 100 chance that the witness correctly identified the cab as blue, and a 17 out of 100 chance that the witness incorrectly identified a green cab as blue. This means there is a 12 + 17 chance out of 100 the witness would identify any cab as blue, regardless of the color.
What is the chance that the witness would identify any cab as blue?

out of 100

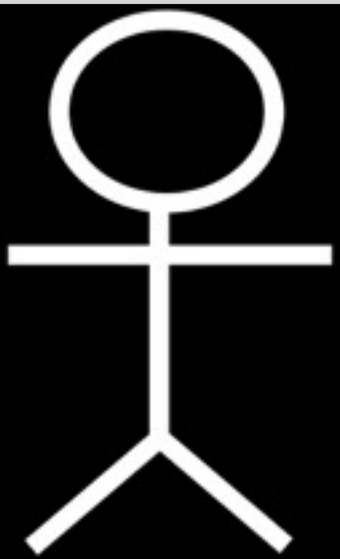

15 Cabs

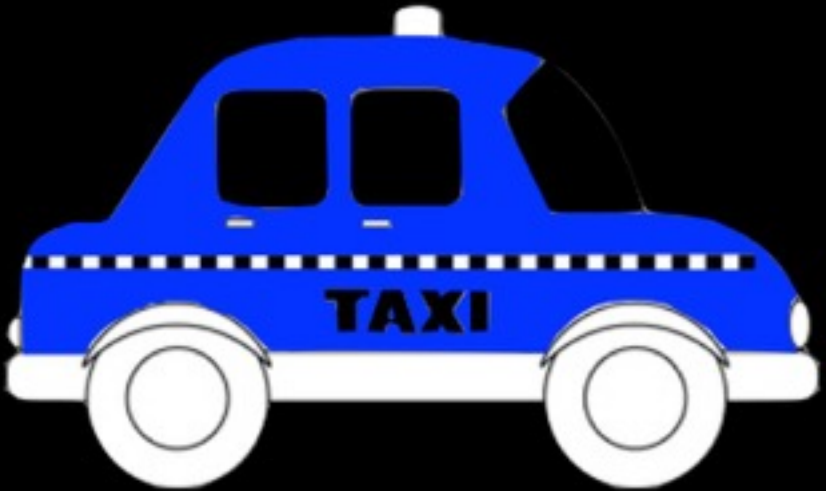

80 correct
20 incorrect

85 Cabs

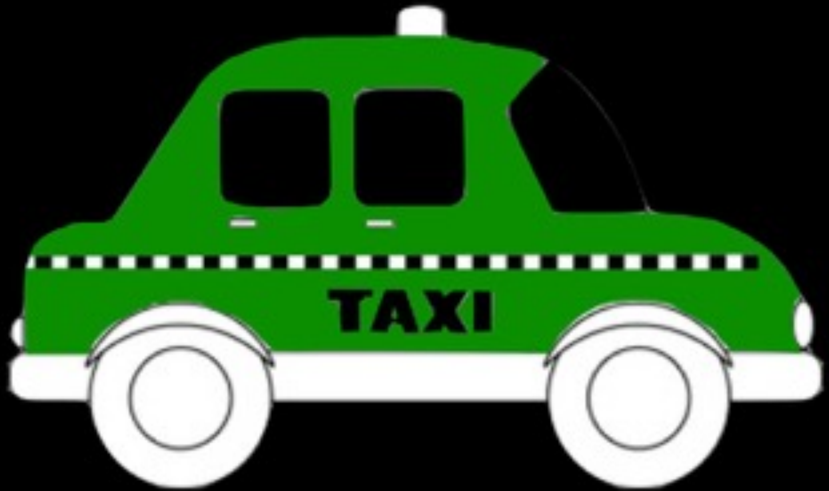

INCORRECT

Now we can use this information to find the chance that the witness correctly identified the cab as blue. To calculate this, we take the chance the witness correctly identified a blue cab as blue (12 out of 100) and divide it by the chance that the witness would identify any cab as blue (29 out of 100).

What is the chance the witness correctly identified the cab as blue?

out of 100

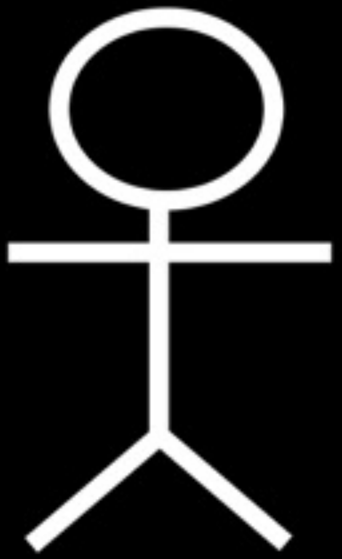

15 Cabs

85 Cabs

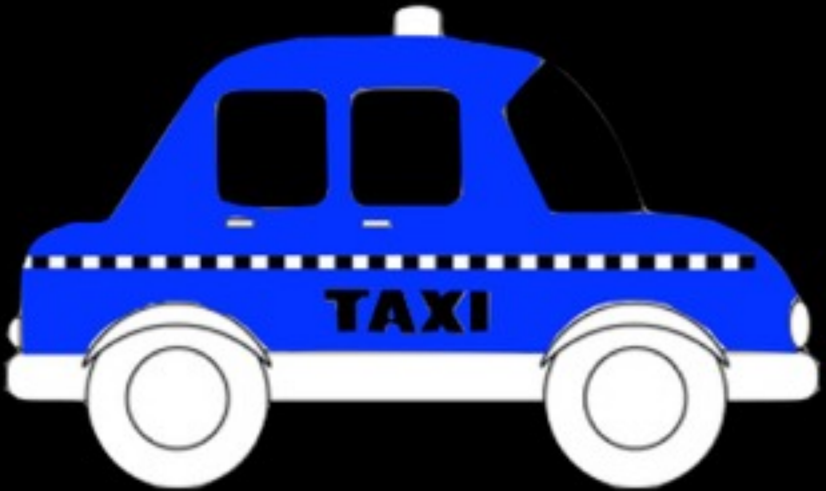

80 correct
20 incorrect

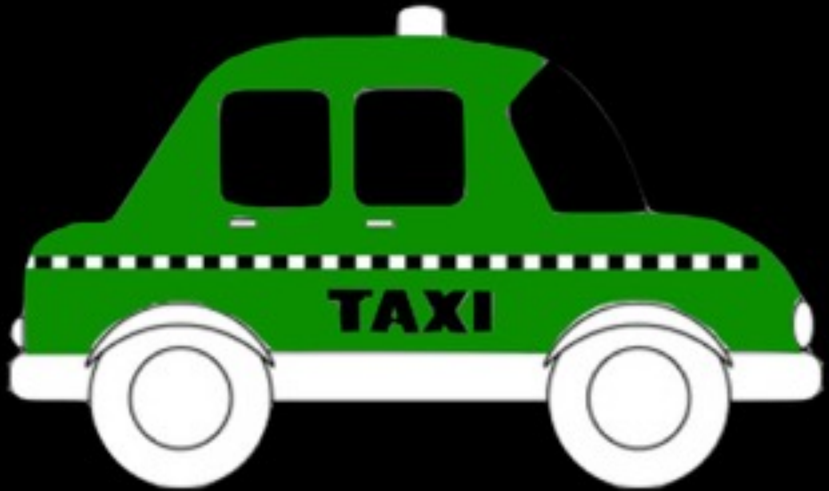

The actual chance of the witness correctly identifying the blue cab as blue is 41%, much smaller than most people (yourself included) originally think. People tend to evaluate the likelihood the witness was correct by only using accuracy information without taking into consideration the relative amount of blue and green cabs in the city.

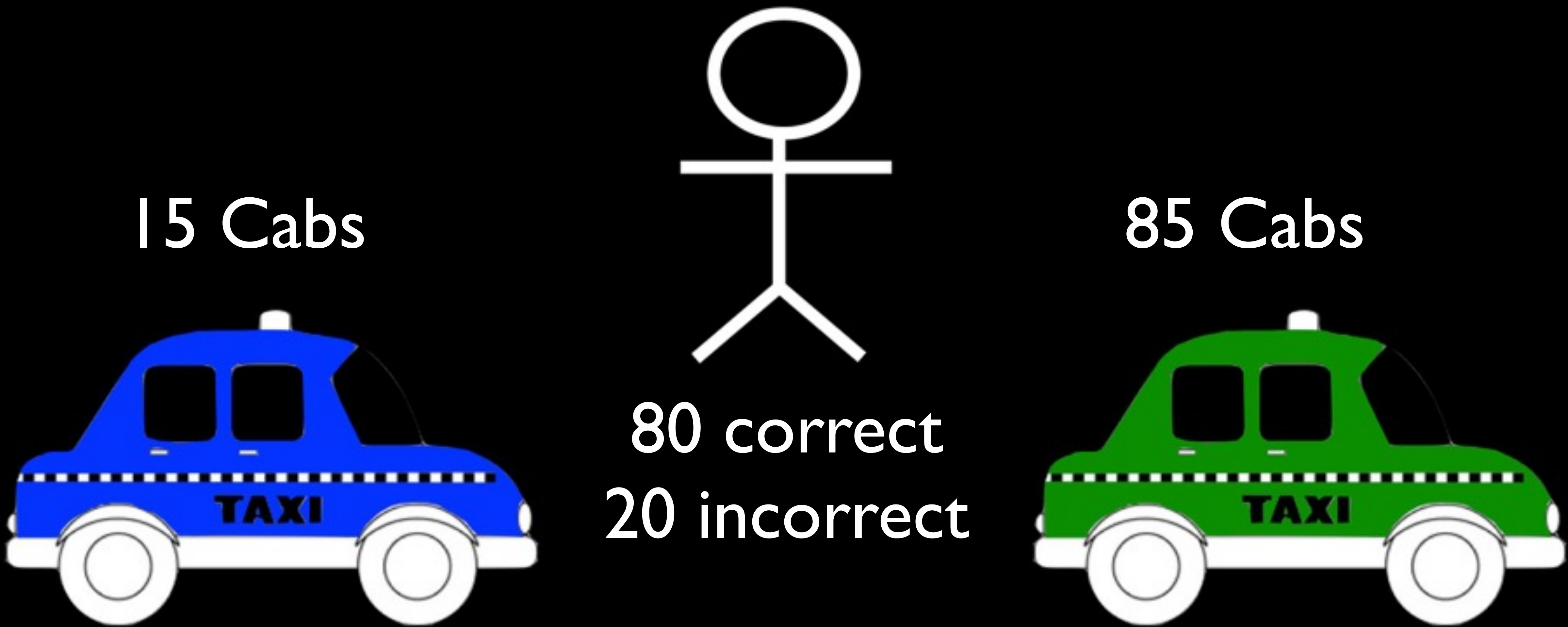

When making judgments we often fail to take into account all the relevant information we’re given, focusing instead on one aspect only.

FEEDBACK FOR INCORRECT RESPONSES

RETURN

Your guess should be a number between 0 and 100. With that in mind, given that the witness identified the cab involved in the accident as blue, what do you think the chance is that it was blue, not green?

out of 100

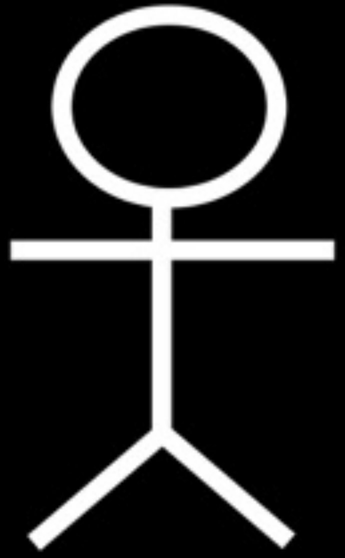

15 Cabs

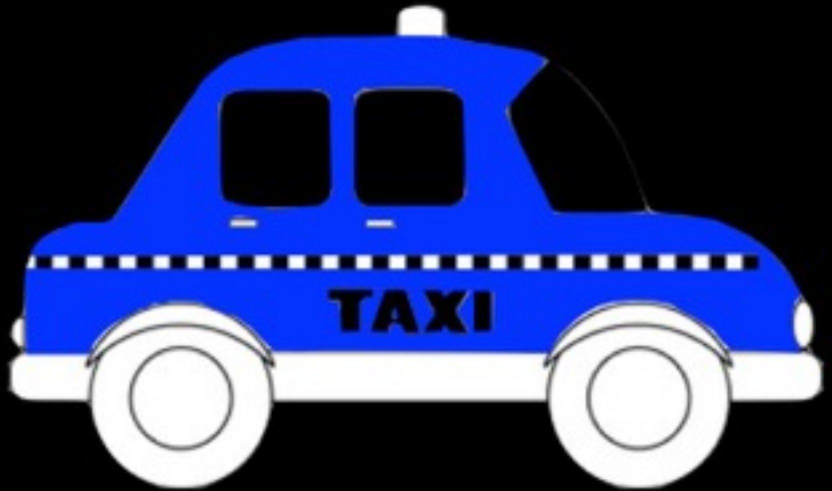

80 correct
20 incorrect

85 Cabs

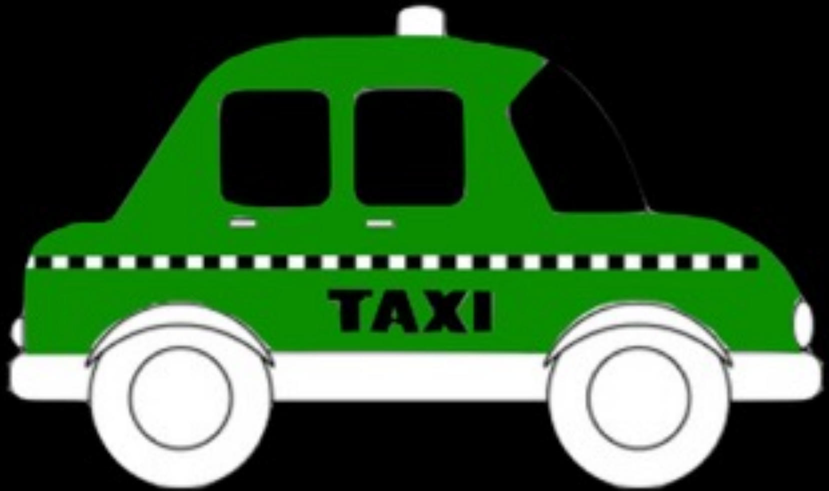

RETURN

Remember, the chance that the witness correctly identified the blue cab as blue is equal to the chance that the cab was blue (15 out of 100) times the chance that the witness was correct (80 out of 100). This means that there was a 15/100 * 80/100 = 12/100 chance the witness correctly identified a blue cab as blue.

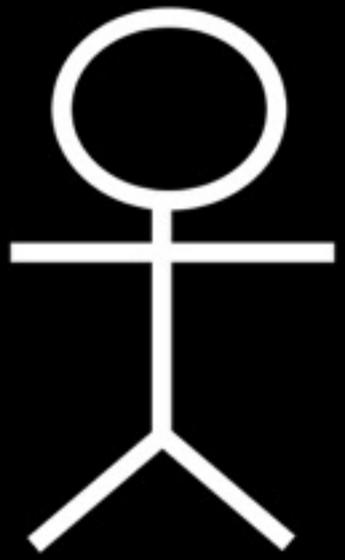

15 Cabs

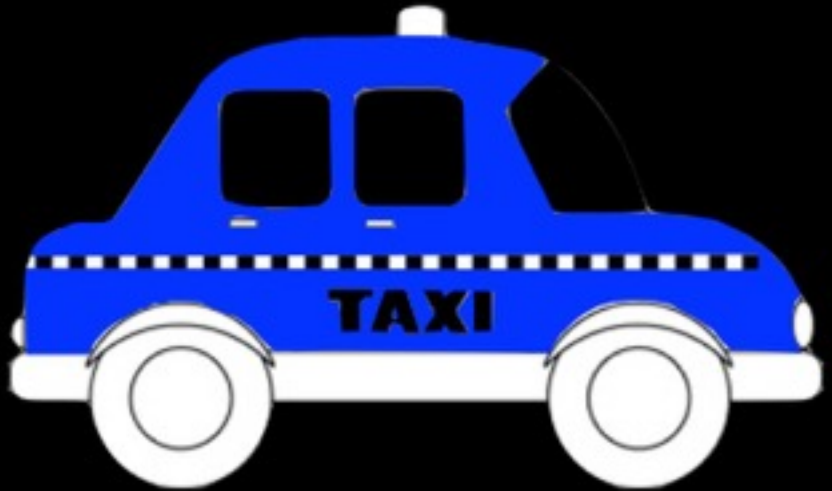

80 correct
20 incorrect

85 Cabs

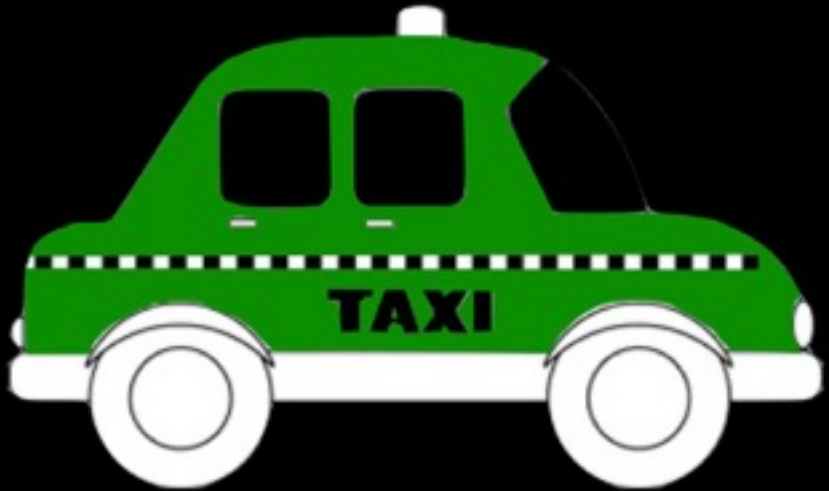

RETURN

The chance that the witness incorrectly identified a green cab as blue is equal to the chance that the cab was green (85 out of 100) times the chance the witness was incorrect (20 out of 100). Therefore, there is an 85 / 100 * 20 / 100 = 17 / 100 chance that the witness incorrectly identified a green cab as blue.

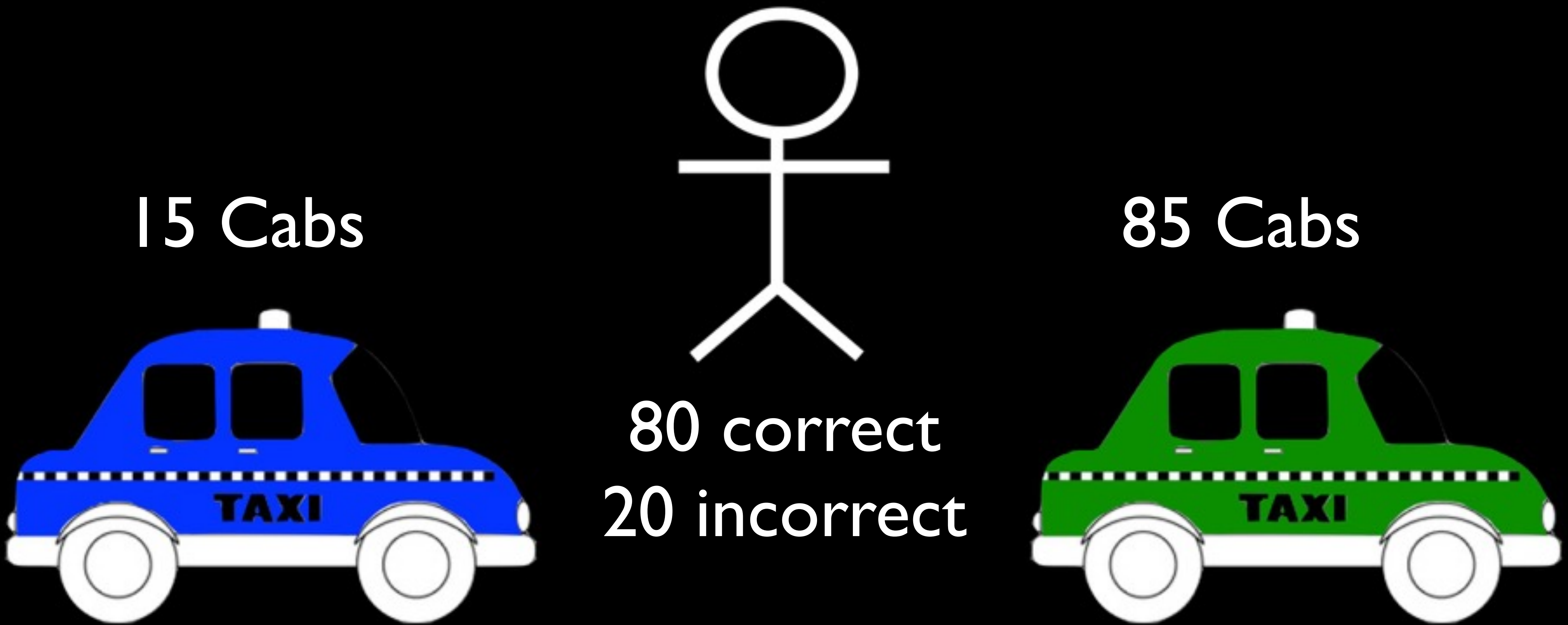

RETURN

The chance that the witness identified any cab as blue regardless of the color is equal to the chance that he identified a blue cab as blue (12 out of 100) plus the chance that he identified a green cab as blue (17 out of 100). This means there is a 12 + 17 = 29 out of 100 chance that the witness would identify any cab as blue.

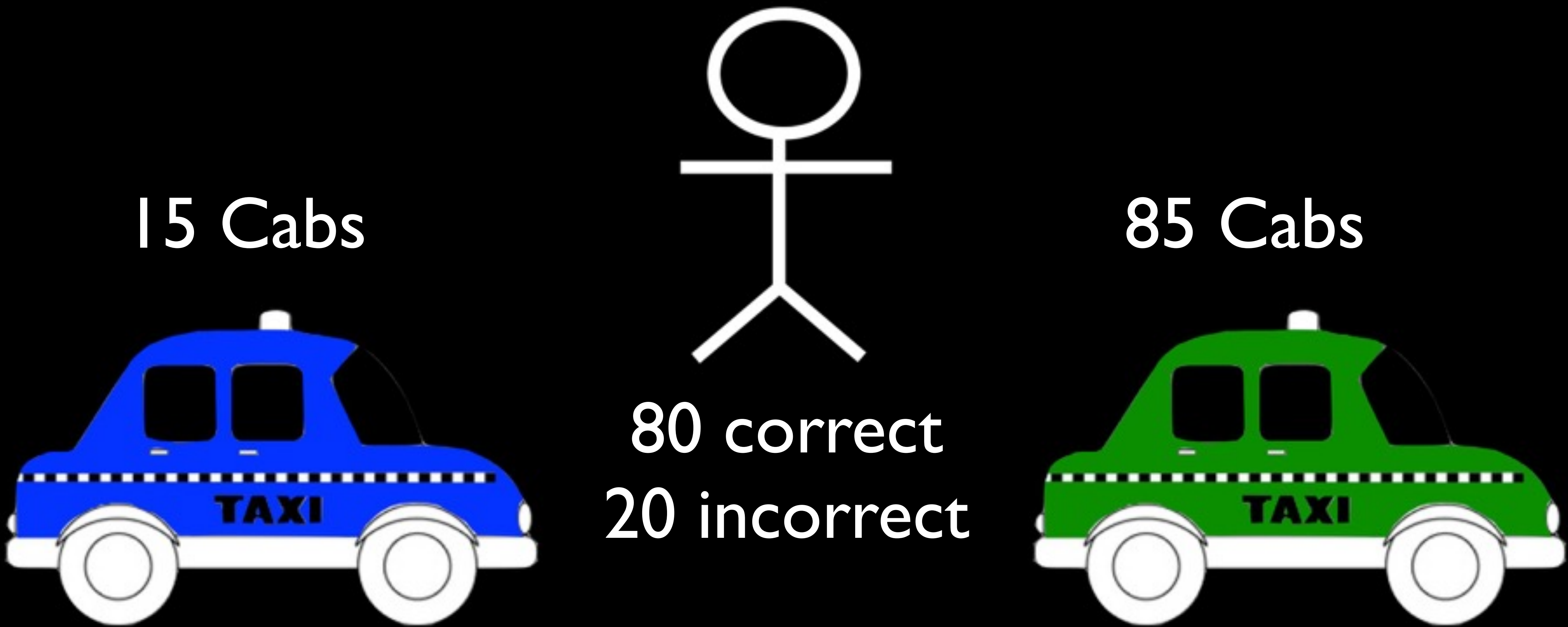

RETURN

The actual chance the witness correctly identified the cab involved in the hit and run as blue is 12 out of 100 (the chance the witness identified a blue cab as blue) divided by 29 out of 100 (the chance the witness identified any cab as blue) = 41 out of 100.

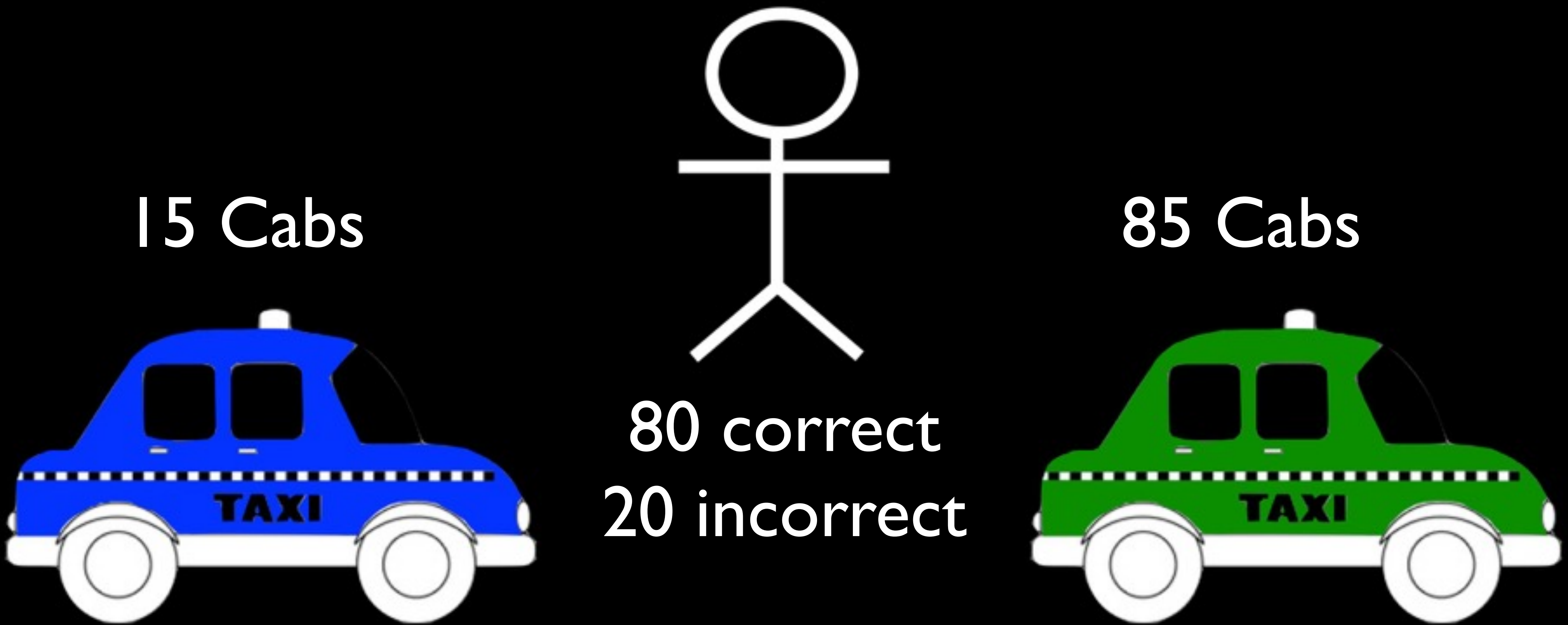

Supplement: S1 Text — (PDF) [file pone.0228782.s001.pdf]
