# Supplementary material for: Learning about the Ellsberg Paradox reduces, but does not abolish, ambiguity aversion
Source: PLoS One. 2020 Mar 4;15(3):e0228782. doi: 10.1371/journal.pone.0228782 (PMC7055742; doi:10.1371/journal.pone.0228782)
Supplement: S2 Text — (DOCX) [file pone.0228782.s002.docx]

**S2 Text. Analysis of ambiguity attitude including trials in which the ambiguous lottery offered $5.**

To verify that the exclusion of $5 trials did not bias the results, we repeated the estimation of ambiguity attitudes (correcting for risk attitudes) using all the trials.

Participants were ambiguity averse before intervention (*n* = 119, *M* = -0.34, *SD* = 0.18) as revealed by a one-sample t-test of ambiguity attitude compared to 0 (indicating ambiguity neutrality), *t*(118) = -20.9, *p* < 0.001. A one-way ANOVA of the pre-intervention ambiguity attitude (Fig A, dark violin plots), with intervention method as a between-subject factor, showed no group difference in baseline ambiguity attitude (AC: *n* = 40, *M* = -0.35, *SD* = 0.16; NC: *n* = 40, *M* = -0.34, *SD* = 0.19; control: *n* = 39, *M* = -0.34, *SD* = 0.19; *F*(2,116) = 0.0960, *p* = 0.909, *η2* = 0.00165). Paired Sign tests comparing post- and pre-intervention ambiguity attitudes within groups showed that participants chose ambiguous options more after the AC (*S* = 28, *p* < 0.05) and NC (*S* = 29, *p* < 0.05) interventions, and marginally after control intervention (*S* = 12, *p* = 0.0711; Bonferroni corrected for three comparisons). To quantify the de-biasing effect of each intervention method, we calculated the difference between the post- and pre-intervention ambiguity attitudes (i.e. the increase in risk-controlled ambiguous choices after intervention). A one-way ANOVA of this difference, with intervention method as a between-subject factor, revealed a significant effect (*F*(2,116) = 10.3, *p* < 0.001, *η^2^* = 0.151 ; Fig B). Post-hoc comparisons further showed that the AC (*M* = 0.12, *SD* = 0.19) and NC (*M* = 0.094, *SD* = 0.17) interventions both decreased participants’ ambiguity aversion compared with control (*M* = -0.043, *SD* = 0.15; FDR adjusted *p*’s < 0.001). The post-hoc comparison also showed that the difference between AC and NC was not significant (FDR adjusted *p* = 0.494), indicating that de-biasing strategies based on either active learning or passive learning performed equally well in our experiment. Non-parametric Mann-Whitey tests of pair-wise intervention group comparison also showed the same results: the increase of ambiguity tolerance was greater in AC compared to control (*U* = 1218, *p* < 0.001), and in NC compared to control (*U* = 1154, *p* < 0.001), but there was no difference between AC and NC (*U* = 806, *p* = 1; Bonferroni corrected for three comparisons). Thus, these results are consistent with the results presented in the main text.


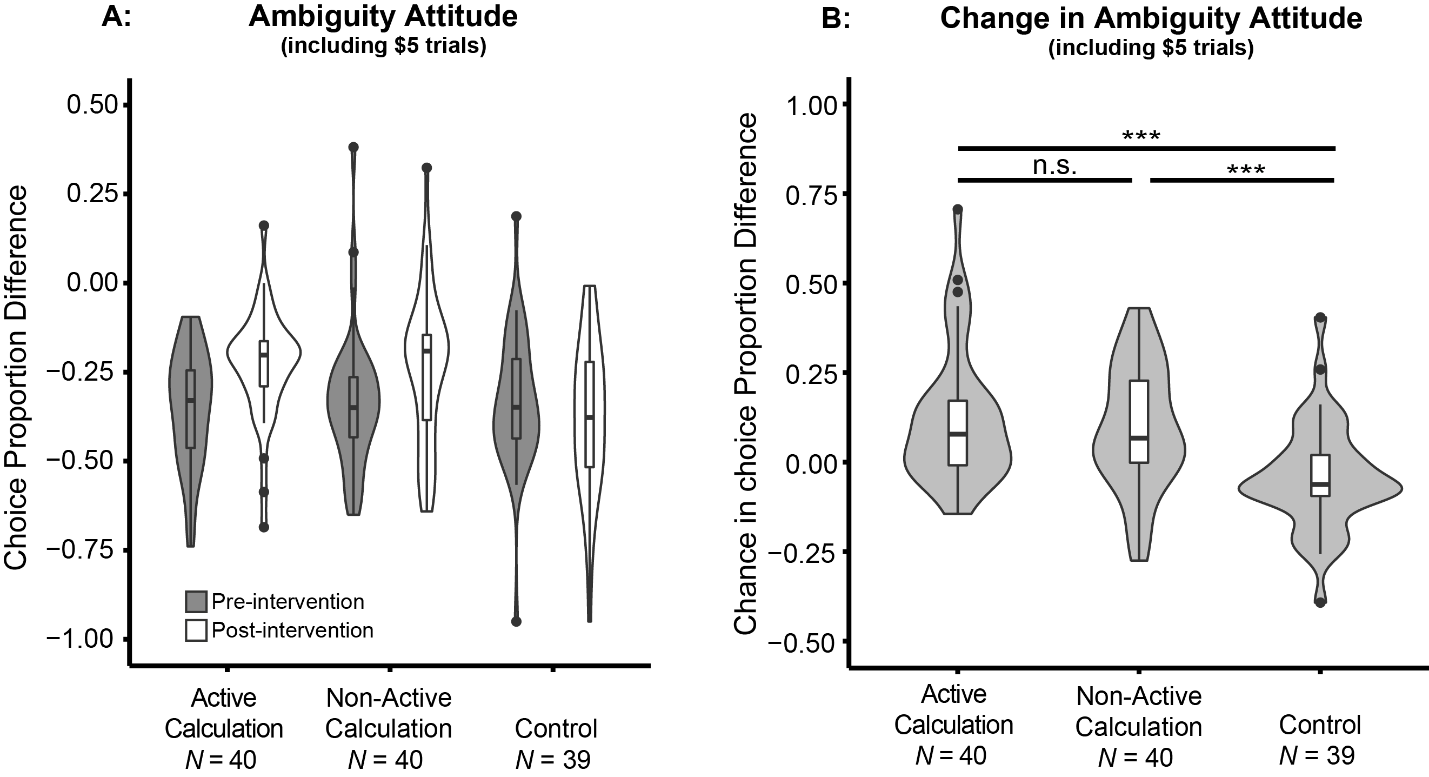


**Fig. Change in ambiguity attitude (including $5 trials) after intervention.** Violin and box plots of: (A) Ambiguity attitude, calculated as the difference between the choice proportion of ambiguous lotteries and the modeled choice proportion of 50% risky lotteries, before and after intervention for each group: Active Calculation, Non-active Calculation, and control. (B) Change in ambiguity attitude after intervention for each group. Positive numbers indicate increase in choice proportion (decrease of ambiguity aversion). *P* values were adjusted by FDR procedures, and significance levels are labeled as: n.s., non-significant; ***, *p* < 0.001. Plots were trimmed within the range of the data. Box plots show the medians with horizontal thick lines. The lower and upper hinges correspond to the first and third quartiles, and the whiskers extend from the hinge to the largest value no further than 1.5 inter-qualitle range (distance between the first and third quartiles) of the data. Outliers beyond the whiskers are plotted by individual dots. Violin plots show the mirrored densities of the data.
