# Supplementary material for: Learning about the Ellsberg Paradox reduces, but does not abolish, ambiguity aversion
Source: PLoS One. 2020 Mar 4;15(3):e0228782. doi: 10.1371/journal.pone.0228782 (PMC7055742; doi:10.1371/journal.pone.0228782)
Supplement: S3 Text — (DOCX) [file pone.0228782.s003.docx]

**S3 Text. Model-free analysis showed that AC and NC interventions reduced ambiguity aversion.**

Besides controlling for risk attitude by the model-based approach, we also looked at change in ambiguity attitude accounting for risk attitude without using behavioral modeling. We calculated the choice proportion of the ambiguous lottery for different ambiguity levels separately (24%, 50%, and 74%), and computed the difference between these proportions at different ambiguity levels (50% vs. 24%, and 74% vs. 24%). Since the influence of risk attitude on the participant’s choices should not depend on the level of ambiguity, it would be cancelled out in these comparisons.

The comparisons between these differences before and after intervention in each group are shown in Fig. A two-way ANOVA on choice difference between 50% and 24% ambiguity levels (Fig A) with phase (pre- and post- intervention) as a within-subject factor and intervention method as a between-subject factor revealed a significant interaction effect, *F*(2,116) = 5.36, *p* < 0.01, *η^2^* = 0.0229. Post-hoc comparisons showed that both AC (pre-intervention, *M* = -0.11, *SD* = 0.14; post-intervention, *M* = -0.062, *SD* = 0.15) and NC (pre-intervention, *M* = -0.11, *SD* = 0.13; post-intervention, *M* = -0.035, *SD* = 0.088) interventions increased the difference between choice proportions of lotteries with 50% and 24% ambiguity levels (AC: FDR adjusted *p* < 0.05, NC: FDR adjusted *p* < 0.001), but not the control intervention (pre-intervention, *M* = -0.063, *SD* = 0.13; post-intervention, *M* = -0.086, *SD* = 0.15; control: FDR adjusted *p* = 0.302; comparing interaction effects between AC and control, FDR adjusted *p* < 0.05). There was no difference in this increase effect between AC and NC groups (FDR adjusted *p* = 0.461). Similarly, a two-way ANOVA on choice difference between 74% and 24% ambiguity levels (Fig B) with phase (pre- and post- intervention) as a within-subject factor and intervention method as a between-subject factor revealed a significant interaction effect, *F*(2,116) = 8.51, *p* < 0.001, *η^2^* = 0.0373. Post-hoc comparisons showed that both AC (pre-intervention, *M* = -0.28, *SD* = 0.23; post-intervention, *M* = -0.12, *SD* = 0.19) and NC (pre-intervention, *M* = -0.30, *SD* = 0.24; post-intervention, *M* = -0.16, *SD* = 0.24) interventions increased the difference between choice proportions of lotteries with 74% and 24% ambiguity levels (FDR adjusted *p*’s < 0.001), but not the control intervention (pre-intervention, *M* = -0.25, *SD* = 0.25; post-intervention, *M* = -0.30, *SD* = 0.26; control: FDR adjusted *p* = 0.254; comparing interaction effects between AC and control FDR adjusted *p* < 0.001). There was no difference in this increase effect between AC and NC interventions (FDR adjusted *p* = 0.760). These results both indicated that AC and NC interventions, but not the control intervention, reduced the effect of ambiguity on participants’ choices.


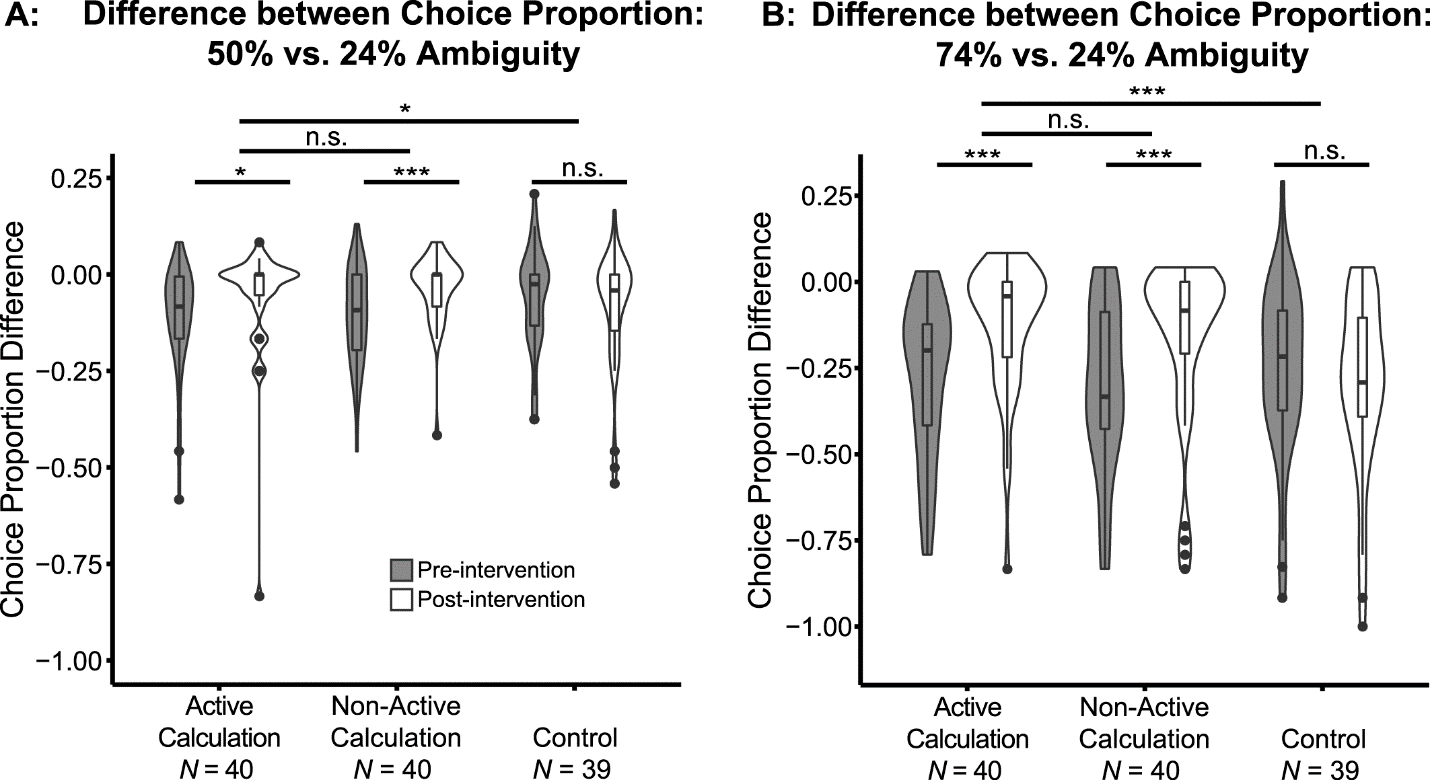


**Fig. The effect of ambiguity level on choice.** Violin and box plots of: (A) difference between choice proprotion of lotteries with 50% and 24% ambiguity levels, a two-way ANOVA with phase (pre- and post- intervention) as the within-subject factor and intervention method as the between-subject factor revealed a significant interaction effect, *F*(2,116) = 5.36, *p* < 0.01, *η^2^* = 0.0229; (B) difference between choice proprotion of lotteries with 74% and 24% ambiguity levels, a two-way ANOVA with phase (pre- and post- intervention) as the within-subject factor and intervention method as the between-subject factor revealed a significant interaction effect, *F*(2,116) = 8.51, *p* < 0.001, *η^2^* = 0.0373. Post-hoc tests were conducted to compare pre- and post- intervention difference within each group, and the difference of this difference bewteen groups, and were shown on the graphs. P values were adjusted by FDR procedure, and siginificance levels are labeled as: n.s., non-significant; *, p < 0.05; **, *p* < 0.01; ***, *p* < 0.001. Both results indicated that AC and NC interventions decreased participants’ sensitivity to lottery ambiguity levels. Plots are trimmed within the range of the data. Box plots show the medians with horizontal thick lines. The lower and upper hinges correspond to the first and third quartiles, and the whiskers extend from the hinge to the largest value no further than 1.5 inter-qualitle range (distance between the first and third quartiles) of the data. Outliers beyond the whiskers are marked by individual dots. Violin plots show the mirrored densities of the data.
