# Supplementary material for: Learning about the Ellsberg Paradox reduces, but does not abolish, ambiguity aversion
Source: PLoS One. 2020 Mar 4;15(3):e0228782. doi: 10.1371/journal.pone.0228782 (PMC7055742; doi:10.1371/journal.pone.0228782)
Supplement: S4 Text — (DOCX) [file pone.0228782.s004.docx]

**S4 Text. Reaction times did not differ across intervention methods.**

We measured participants’ reaction time (RT) as the duration between the onset of lottery presentation and the time of button press, thus it reflected the duration of the whole decision making process. Because ambiguous trials were more complex than risky lotteries, participants might have spent more time on those trials. Also, as the intervention taught participants that the best strategy in ambiguous trials is to always choose the ambiguous lottery, we would expect faster responses in ambiguous trials after the intervention. We calculated the averaged reaction time for risky trials and ambiguous trials, separately for pre- and post- intervention choices for each of the three groups (Table). We excluded the trials in which the varying lottery was $5, as in the analysis of ambiguity and risk attitudes.

**Table. Descriptive statistics of reaction time (ms) in ambiguous and risky trials, before and after intervention for the three intervention groups, Mean (Standard deviation).**

|  | **Active Calculation** | **Non-active Calculation** | **Control** |
| --- | --- | --- | --- |
| Number of participants | 40 | 40 | 39 |
|  |  |  |  |
| Ambiguous trials (pre-intervention) | 2425 (598) | 2396 (547) | 2413 (703) |
|  |  |  |  |
| Ambiguous trials (post-intervention) | 1654 (462) | 1709 (466) | 1814 (512) |
|  |  |  |  |
| Risky trials (pre-intervention) | 2476 (595) | 2419 (524) | 2434 (691) |
|  |  |  |  |
| Risky trials (post-intervention) | 1741 (476) | 1828 (439) | 1822 (533) |

We first looked at whether there was a difference between RTs in ambiguous and risky trials. In pre-intervention choices, a two-way ANOVA on averaged RT with uncertainty type (risky or ambiguous) as a within-subject factor and intervention method as a between-subject factor revealed a main effect of uncertainty, *F*(1,116) = 6.46, *p* < 0.05, *η^2^* = 0.000677, suggesting that participants were faster in ambiguous trials (*Mean* = 2411, *SD* = 613, *N* = 119) than in risky trials (*Mean* = 2443, *SD* = 602, *N* = 119). There was no main effect of intervention, *F*(2,116) = 0.0509, *p* = 0.950, *η^2^* = 0.000867, nor interaction effect between uncertainty type and intervention, *F*(2,116) = 0.599, *p* = 0.551, *η^2^* = 0.000126. Similarly, in post-intervention choices, a two-way ANOVA on averaged RT with uncertainty type (risky or ambiguous) as a within-subject factor and intervention method as a between-subject factor revealed a main effect of uncertainty, *F*(1,116) = 11.4, *p* < 0.01, *η^2^* = 0.00562, suggesting that participants were faster in ambiguous trials (*Mean* = 1725, *SD* = 481, *N* = 119) than in risky trials (*Mean* = 1797, *SD* = 481, *N* = 119). There was no main effect of intervention, *F*(2,116) = 0.662, *p* = 0.518, *η^2^* = 0.0106, nor interaction effect, *F*(2,116) = 2.39, *p* = 0.0958, *η^2^* = 0.00237. These results suggest that participants are generally faster in making decisions in ambiguous trials, in both pre-intervention and post-intervention choices, opposite to the hypothesis that the complexity of ambiguous trials would make participants spend more time making decisions. But intervention methods do not influence the difference in decision making speed between ambiguous and risky trials.

­We next looked at whether intervention influenced participants’ decision making speed in ambiguous trials (Fig) after participants learned the Ellsberg Paradox. A two-way ANOVA on averaged RT of ambiguous trials with phase (pre- or post- intervention) as a within-subject factor and intervention method as a between-subject factor revealed a main effect of phase, *F*(1,116) = 373, *p* < 0.001, *η^2^* = 0.282, but no main effect of intervention, *F*(2,116) = 0.225, *p* = 0.799, *η^2^* = 0.00340. There was no interaction effect between phase and intervention method, *F*(2,116) = 1.96, *p* = 0.146, *η^2^* = 0.00411. We also investigated intervention’s effect on RT in risky trials (Fig B), and a two-way ANOVA on averaged RT of risky trials with phase (pre- or post- intervention) as a within-subject factor and intervention method as a between-subject factor revealed a main effect of phase, *F*(1,116) = 371, *p* < 0.001, *η^2^* = 0.262, but no main effect of intervention, *F*(2,116) = 0.0155, *p* = 0.985, *η^2^* = 0.000238. There was no interaction effect between phase and intervention methods, *F*(2,116) = 1.81, *p* = 0.169, *η^2^* = 0.00345. These restults suggest that participants generally became faster following the intervention, regardless of intervention method, most likely as a result of familiarity with the task.


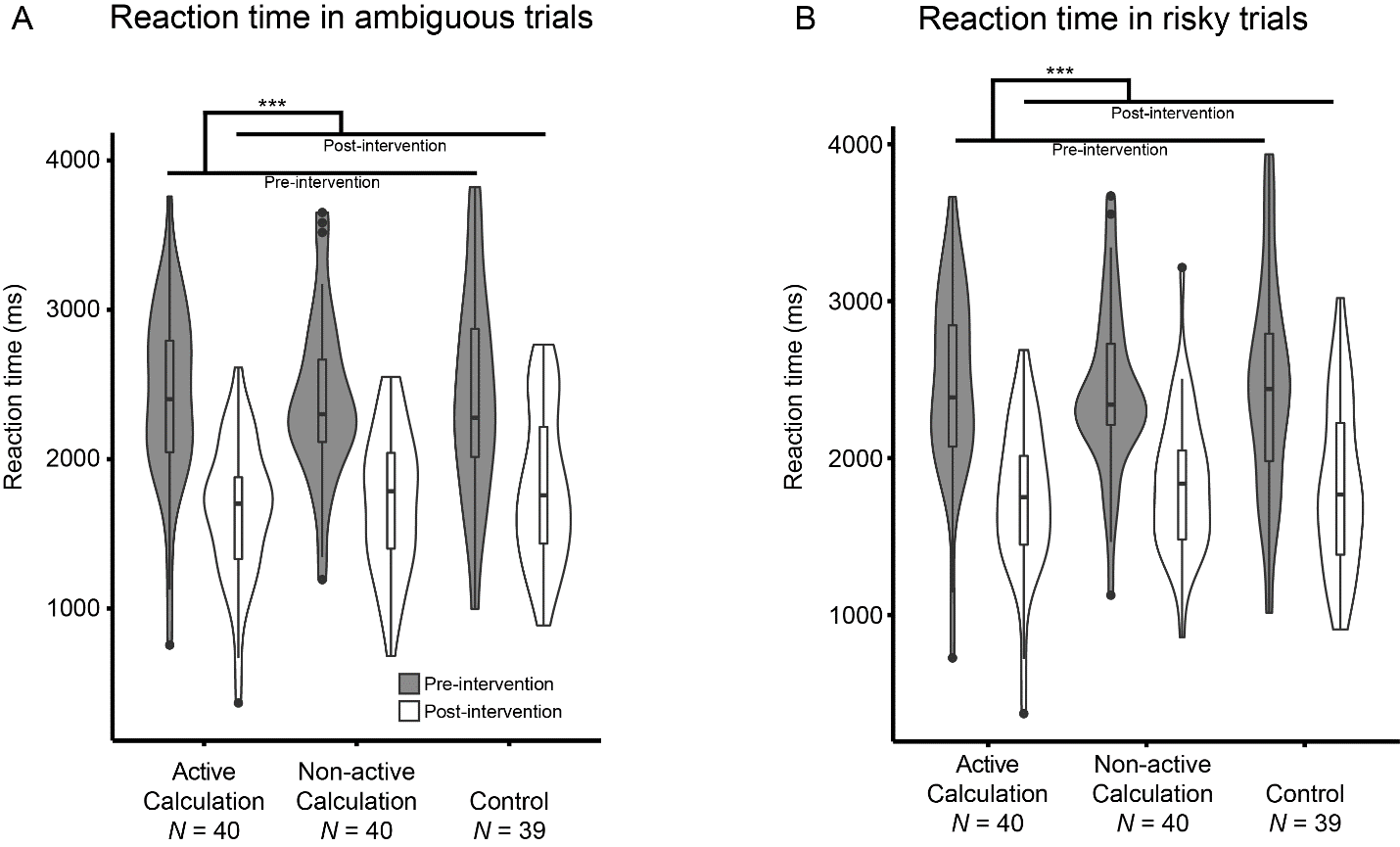


**Fig. Reaction time (RT) in ambiguous and risky trials.** Violin and box plots of: (A) Averaged RT in ambiguous trials, before and after the intervention for each group: Active Calculation, Non-active Calculation, and control. (B) Averaged reaction time in risky trials, before and after the intervention for each group: Active Calculation, Non-active Calculation, and control. Significance levels were labeled as: *** indicating *p* < 0.001. Plots are trimmed within the range of the data. Box plots show the medians with horizontal thick lines. The lower and upper hinges correspond to the first and third quartiles, and the whiskers extend from the hinge to the largest value no further than 1.5 inter-qualitle range (distance between the first and third quartiles) of the data. Outliers beyond the whiskers are plotted by dots individually. Violin plots show the mirrored densities of the data.
