# Supplementary material for: Learning about the Ellsberg Paradox reduces, but does not abolish, ambiguity aversion
Source: PLoS One. 2020 Mar 4;15(3):e0228782. doi: 10.1371/journal.pone.0228782 (PMC7055742; doi:10.1371/journal.pone.0228782)
Supplement: S1 Table — One-way ANOVA of change in ambiguity attitude after intervention, with intervention method as between-subject factor. Ambiguity attitude was calculated as the difference between the ambiguous lottery choice proportion and the modeled 50% risky lottery choice proportion. (DOCX) [file pone.0228782.s005.docx]

**S1 Table. ANOVA of change in model-based ambiguity attitude after intervention.**

| *Source* | *df* | *SS (type III)* | *MS* | *F* | *η^2^* | *p* |
| --- | --- | --- | --- | --- | --- | --- |
| Intervention method | 2 | 0.794 | 0.397 | 11.5 | 0.165 | 2.78×10^-5 ***^ |
| Subject | 116 | 4.00 | 0.0345 |  |  |  |

Significance level, * *p* < 0.05, ** *p* < 0.01, *** *p* < 0.001

One-way ANOVA of change in ambiguity attitude after intervention, with intervention method as between-subject factor. Ambiguity attitude was calculated as the difference between the ambiguous lottery choice proportion and the modeled 50% risky lottery choice proportion.
