# Supplementary material for: Learning about the Ellsberg Paradox reduces, but does not abolish, ambiguity aversion
Source: PLoS One. 2020 Mar 4;15(3):e0228782. doi: 10.1371/journal.pone.0228782 (PMC7055742; doi:10.1371/journal.pone.0228782)
Supplement: S4 Table — 113 participants with a score of understanding the Ellsberg Paradox were included in this analysis. The result confirms the interaction effect between phase and interaction method, that participants in both AC and NC groups increased choice of the varying risky lottery compared with the Control group. AC and NC groups did not show difference in this effect (χ2 = 0.0803, p = 0.777). (DOCX) [file pone.0228782.s008.docx]

**S4 Table. Summary of all effects from logistic regression of choices of the varying risky lotteries.**

| *Effect Name* | *Beta Estimate* | *Standard Deviation* | *Z-scored Beta* | *p* |
| --- | --- | --- | --- | --- |
| (Intercept) | 0.320 | 0.819 | 0.391 | 0.696 |
|  |  |  |  |  |
| Phase: post-intervention – pre-intervention | -0.104 | 0.170 | -0.611 | 0.541 |
|  |  |  |  |  |
| Intervention: AC - Control | 0.127 | 0.830 | 0.153 | 0.879 |
|  |  |  |  |  |
| Intervention: NC - Control | -0.000772 | 0.837 | -0.00092 | 0.999 |
|  |  |  |  |  |
| Risk level | 1.58 | 0.0323 | 48.9 | < 10^-15 ***^ |
|  |  |  |  |  |
| Value | 1.33 | 0.0304 | 43.8 | < 10^-15 ***^ |
|  |  |  |  |  |
| EP score | 0.00804 | 0.875 | 0.00918 | 0.993 |
|  |  |  |  |  |
| Age | -0.365 | 0.185 | -1.97 | 0.0483 ^*^ |
|  |  |  |  |  |
| Gender: Female - Male | -0.331 | 0.340 | -0.973 | 0.331 |
|  |  |  |  |  |
| Phase × Intervention: AC - Control | 0.618 | 0.248 | 2.49 | 0.0127 ^*^ |
|  |  |  |  |  |
| Phase × Intervention: NC - Control | 0.549 | 0.238 | 2.31 | 0.0209 ^*^ |
|  |  |  |  |  |
| EP score × Intervention: AC - Control | -0.0323 | 0.929 | -0.0348 | 0.972 |
|  |  |  |  |  |
| EP score × Intervention: NC - Control | -0.540 | 0.925 | -0.584 | 0.559 |

Significance level, * *p* < 0.05, ** *p* < 0.01, *** *p* < 0.001

113 participants with a score of understanding the Ellsberg Paradox were included in this analysis. The result confirms the interaction effect between phase and interaction method, that participants in both AC and NC groups increased choice of the varying risky lottery compared with the Control group. AC and NC groups did not show difference in this effect (χ^2^ = 0.0803, *p* = 0.777).
